# Gut microbiota: new links between exercise and disease

**DOI:** 10.3389/fmicb.2026.1746359

**Published:** 2026-01-30

**Authors:** Yini Wu, Yinfeng Wang, Qingtong Zhang, Lijuan Yao, Zhennan Ma, Leqin Chen

**Affiliations:** 1 China Institute of Sport Science, Beijing, China; 2 School of Physical Education, Shanxi Normal University, Taiyuan, Shanxi, China

**Keywords:** aerobic exercise, gut microbiota, immune diseases, metabolic diseases, moderate-intensity exercise, neurological diseases

## Abstract

As the “second genome” of the human body, the intestinal microbiota plays a key role in preventing the onset and progression of obesity, metabolic disorders, and inflammatory diseases by modulating immune function, maintaining metabolic homeostasis, and reinforcing mucosal barrier integrity. This review systematically investigates the biological and physiological mechanisms underlying the interaction between exercise and the gut microbiota in disease prevention. Existing evidence suggests that exercise, as a non-pharmacological intervention, can prevent and manage obesity, diabetes, and neurodegenerative diseases by reshaping the composition and function of the gut microbiota, suppressing oxidative stress, reducing inflammatory markers, and maintaining intestinal mucosal barrier homeostasis. Current evidence has begun to elucidate the molecular mechanisms by which the gut microbiota mediates disease prevention and progression under varying exercise intensities, modalities, and durations. However, the structural and functional changes of the gut microbiota induced by different exercise doses remain insufficiently characterized, limiting the ability to establish clear exercise-dose relationships for disease prevention. This article systematically reviews the fundamental characteristics of the gut microbiota and the physiological mechanisms underlying exercise intervention in disease prevention through the microbiota, with a focus on exploring the interaction network among the microbiota, exercise, and disease states. Although exercise-induced regulation of the gut microbiota and its metabolites, including short-chain fatty acids (SCFAs), tryptophan metabolites, and bile acids, has demonstrated adaptive and regulatory advantages in disease prevention, the specific effects of exercise-driven changes in the microbiota on various diseases still require extensive experimental validation. In the future, greater attention should be given to the differential effects of varying exercise doses on individual gut microbiota profiles, as well as the long-term impact of exercise-modulated gut microbiota on disease outcomes. On this basis, novel therapeutic strategies should be proposed to promote the enrichment of exercise-responsive microbial populations and harness the protective potential of the gut microbiota for disease prevention.

## 1 Introduction

The intestinal microbiota, as a key determinant of intestinal health, also functions as an invisible microbial regulator of overall human health, maintaining a mutually dependent and adaptive relationship with the host. Due to the beneficial effects of the gut microbiota and its metabolites on disease prevention, this field has attracted increasing scientific attention in recent years. The intestinal microbiota and its metabolites participate in host energy utilization and metabolic regulation (Na et al., 2019), synthesize essential vitamins, including vitamin K and B vitamins, thereby contributing to nutrient absorption, promote intestinal mucosal barrier integrity, and enhance immune function (Krawczyk et al., 2021). Conversely, dysbiosis of the intestinal microbiota can induce chronic inflammation, disrupt interorgan coordination, and impair systemic homeostasis, ultimately contributing to disease onset and progression (Siddiqui et al., 2025). Previous studies have demonstrated that genetic background, aging, specific disease states, and pharmacological interventions significantly alter gut microbiota composition (Jacobs and Braun, 2014; Coman and Vodnar, 2020; Chen et al., 2021), whereas long-term dietary patterns are regarded as a primary factor shaping intestinal microbiota structure. A diet rich in dietary fiber promotes the proliferation of beneficial microorganisms, whereas a diet high in sugar and fat disrupts intestinal microecological balance (Randeni et al., 2024). Although diet is widely regarded as a major determinant of gut microbiota composition and is supported by extensive experimental evidence, longitudinal data examining the effects of exercise interventions on the gut microbiota remain limited. Regular physical activity combined with a healthy dietary pattern is widely recommended as an effective strategy for maintaining health. Exercise accelerates intestinal peristalsis, facilitates the colonization of beneficial microorganisms, reduces the accumulation of potentially harmful bacteria, preserves intestinal barrier function, and promotes gut microbiota homeostasis (Monda et al., 2017). As an important lifestyle intervention for health promotion and disease prevention, the benefits of exercise extend beyond improvements in cardiovascular function and muscle strength. Numerous studies have demonstrated a bidirectional regulatory relationship between exercise and the gut microbiota, whereby exercise alters microbial structure and function, while the microbiota and its metabolites influence exercise adaptation, capacity, and performance (Monda et al., 2017). Studies have reported differences in microbial diversity and the abundance of specific bacterial genera between athletes and sedentary individuals. However, these findings remain controversial due to heterogeneity in study populations, variation in exercise modalities, and insufficient control of dietary factors, and the underlying causal mechanisms remain unclear (Tarracchini et al., 2022). Existing literature has explored the mechanisms through which exercise and the gut microbiota influence specific disease processes (2–3, 6, 8–9). This paper integrates evidence on exercise-induced alterations in the gut microbiota across various diseases and examines the regulatory network linking exercise, the gut, and the microbiota from an interorgan interaction perspective. This article reviews current research on the role of exercise-mediated gut microbiota in disease progression, aiming to elucidate how exercise modulates the gut microbiota and how microbiota-derived metabolites influence the gut-organ axis, thereby contributing to disease prevention and delayed progression.

## 2 Methods

### 2.1 Retrieval strategy

Two systematically trained researchers independently searched PubMed, Web of Science, Embase, China National Knowledge Infrastructure (CNKI), Wanfang Data, and the VIP Database. The literature search covered the period from January 1, 2000 to August 1, 2025. For the Chinese databases, searches were conducted using “gut microbiota,” “exercise,” and “disease” as core keywords, combined with terms such as “aerobic exercise,” “anaerobic exercise,” “low-, moderate-, and high-intensity exercise,” as well as diseases of the digestive, nervous, and immune systems. For the English databases, “intestinal flora,” “exercise,” and “disease” were used as subject terms and combined with relevant free-text terms.

### 2.2 Literature screening and inclusion and exclusion criteria

**Figure 1:**
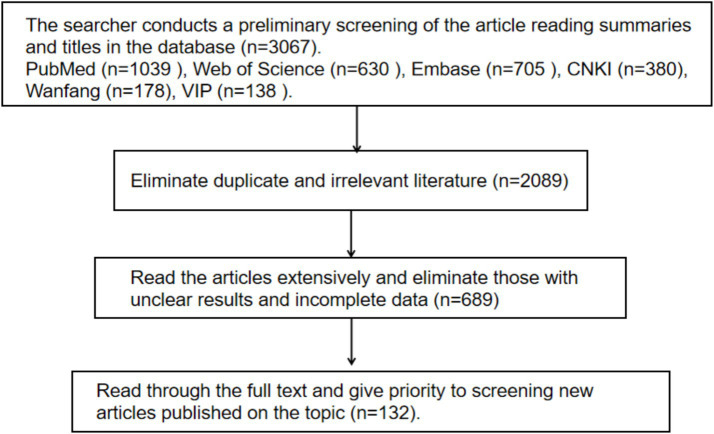
Flow diagram of the literature retrieval strategy.

Two independent researchers conducted an initial search and identified a total of 3,067 records. The inclusion criteria were original research articles or review studies investigating the association between the gut microbiota, exercise interventions, and diseases. The exclusion criteria included duplicate publications, irrelevance to the review topic, incomplete data, unclear results or conclusions, and studies for which the full text was unavailable. After systematic training, the two reviewers independently screened the literature according to the predefined inclusion and exclusion criteria. Any discrepancies were resolved through discussion with a third researcher to reach a consensus. In total, 3,067 records were retrieved, of which 2,089 were excluded, including duplicate publications (532 records), irrelevant studies (1,543 records), and studies without accessible full texts (14 records). After full-text assessment, 689 studies were further excluded, primarily due to unclear results or conclusions (480 studies) and incomplete data (209 studies). Ultimately, 132studies met the inclusion criteria and were included in the review. The literature screening process is illustrated in Figure 1.

## 3 Intestinal flora

**Figure 2:**
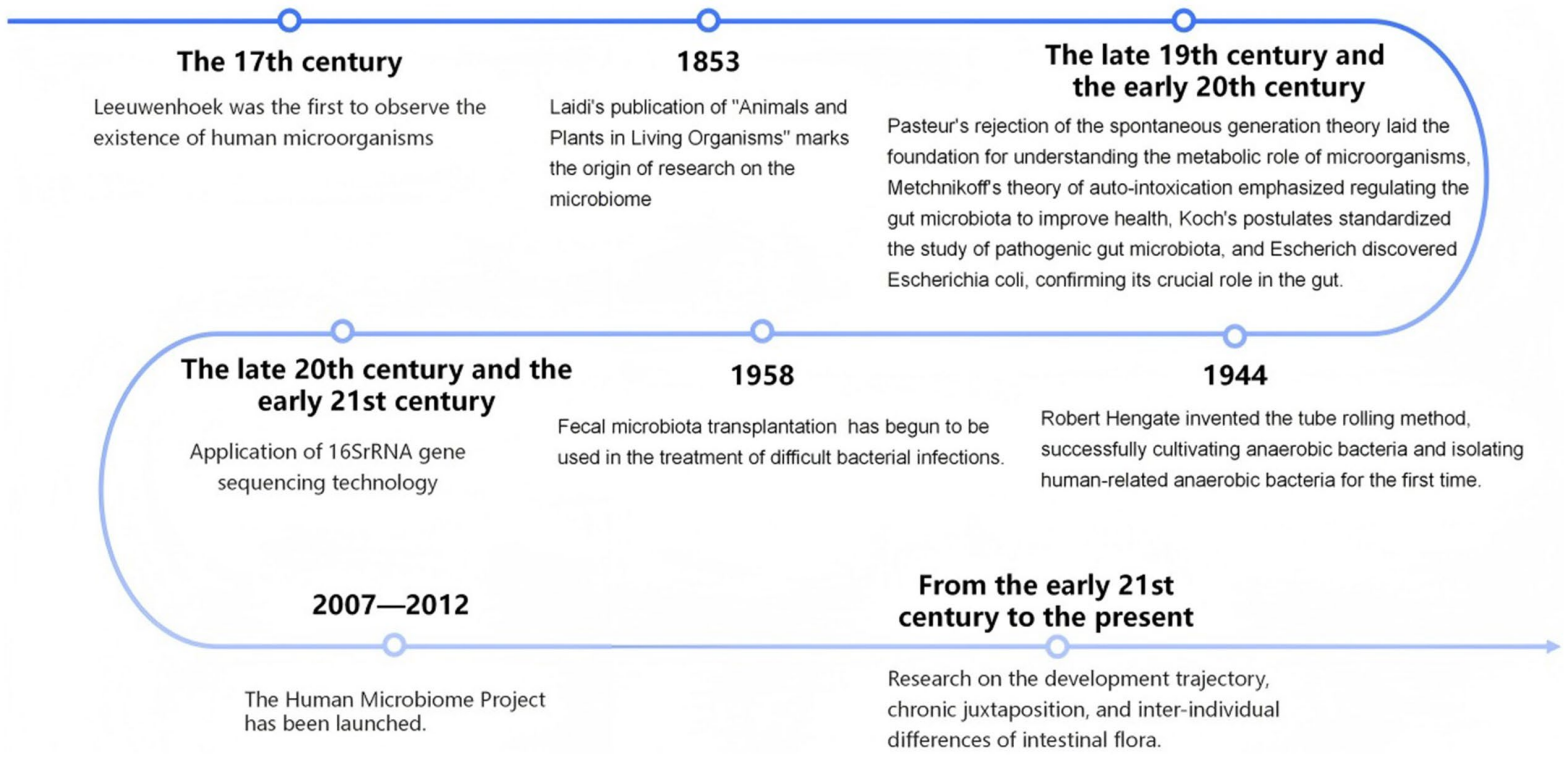
Timeline of gut microbiota development.

The gut microbiota refers to the collective community of microorganisms that colonize the human gastrointestinal tract. In healthy adults, the gut microbiome is predominantly composed of *Firmicutes* and *Bacteroidetes*, with relatively lower proportions of *Actinobacteria*, *Proteobacteria*, and *Bacilli* (Adak and Khan, 2019). Under physiological conditions, the intestinal microbiota regulates host physiological functions through the production of a wide range of metabolites. However, under pathological conditions, microbial dysbiosis reduces the production of beneficial SCFAs, compromises intestinal barrier integrity, and increases the generation of harmful metabolites, including endotoxins, trimethylamine N-oxide, and phenylacetylglutamine. These substances enter the systemic circulation, impair host defense capacity, and contribute to disease-related pathological manifestations (Chen et al., 2019). An overview of recent advances in gut microbiota research is presented in Figure 2.

## 4 Bidirectional regulation between the gut microbiota and exercise

The relationship between the gut microbiota and exercise constitutes a bidirectional regulatory process. Exercise alters the structure and function of the gut microbiota, thereby influencing microbial metabolic pathways and metabolite profiles within the host, and improving host energy metabolism, nutrient absorption and utilization, immune regulation, and neural signaling pathways. The intestinal microbiota can, in turn, influence exercise performance, adaptive responses to exercise, and exercise regulation through the production of metabolites, including SCFAs, bile acids, and neurotransmitters. Collectively, these mechanisms interact to form a closed regulatory network linking exercise, the gut microbiota, and the host.

### 4.1 Exercise performance, exercise adaptation, exercise regulation, and exercise function of intestinal flora

#### 4.1.1 The influence of gut microbiota on athletic performance

The gut microbiota and its metabolites can influence athletic performance through multiple physiological pathways. The gut microbiota ferments dietary fiber into SCFAs which contribute to nutrient digestion and absorption and serve as energy substrates during exercise. Oxidation of SCFAs plays a significant role in energy provision, making this effect more pronounced during prolonged endurance exercise (Giron et al., 2022). Animal studies have shown (Siddharth et al., 2017) that supplementation with butyrate-producing bacteria enhances treadmill exercise endurance and prolongs exercise duration in mice. Gut microbiota–derived metabolites regulate muscle metabolism and function, promote muscle protein synthesis, and facilitate post-exercise muscle repair and growth. Transplantation of fecal microbiota from exercise-trained mice into high-fat diet–fed recipient mice significantly increased skeletal muscle AMP-activated protein kinase phosphorylation, insulin-like growth factor 1 signaling, and glucose uptake, thereby improving muscle metabolism and promoting protein synthesis (Aoi et al., 2023). The gut microbiota indirectly enhances athletic performance by modulating immune function and attenuating inflammatory responses. The relative abundance of Prevotella in the intestines of competitive cyclists is positively correlated with branched-chain amino acid metabolic enzyme activity, and athletes with a high abundance of *Prevotella* exhibit superior athletic performance (Kang et al., 2021). Moderate exercise helps maintain gut microbial balance, enhances intestinal barrier function, reduces endotoxin translocation into the bloodstream, and lowers systemic inflammation, thereby creating a favorable internal environment for exercise. In contrast, gut microbial dysbiosis may increase inflammatory responses and impair muscle function and exercise endurance.

#### 4.1.2 The influence of intestinal Flora on exercise adaptation

During sports training, the body undergoes a series of adaptive changes in which the gut microbiota plays a significant role. The gut microbiota is involved in regulating exercise-induced metabolic adaptations, enhancing insulin sensitivity, and promoting lipid metabolism. The combination of exercise intervention and probiotic supplementation significantly enhanced insulin sensitivity in obese mice, reduced blood glucose levels, and ameliorated lipid metabolic disorders (Ma et al., 2022). Exercise accelerates interactions between the gut microbiota and the immune system, thereby regulating immune cell function and inflammatory cytokine secretion. Exercise-induced secondary bile acids regulate immune cell activity through the farnesoid X receptor, fibroblast growth factor 15/19, and Takeda G-protein-coupled receptor 5 signaling pathways, thereby enhancing the anti-inflammatory macrophage phenotype (Codella et al., 2018). Gut microbiota–derived metabolites, particularly SCFAs, participate in signal transduction, metabolic regulation, and inflammatory processes, thereby modulating energy and lactate metabolism to enhance athletic performance. Compared with sedentary individuals, those who exercise regularly and adhere to specific dietary patterns exhibit significantly distinct gut microbiota compositions (Song and Liao, 2019). After extreme endurance events such as marathons and ultramarathons, the abundance of the intestinal bacterium *Veillonella* increases, which may contribute to enhanced athletic performance. However, not all forms of exercise are beneficial to the gut microbiota, as insufficient energy intake during high-intensity interval training can disrupt gut microbiota integrity and function (Zhang et al., 2025).

#### 4.1.3 The influence of gut microbiota on exercise regulation

The composition and diversity of the gut microbiota are considered key determinants of skeletal muscle metabolism and function. A systematic review reported that sarcopenia is closely associated with distinct fecal microbial communities, and rat models of sarcopenia exhibit significant alterations in fecal microbiota function (Liu et al., 2021). The gut microbiota regulates muscle protein synthesis and degradation, thereby affecting muscle mass and function and modulating microbial gene expression involved in nutrient biosynthesis and metabolism (Siddharth et al., 2017). The gut microbiota influences muscle metabolism through multiple nutrition-sensitive signaling pathways. The mechanistic target of rapamycin and AMP-activated protein kinase are key nutrition-sensitive metabolic regulators that are essential for maintaining cellular homeostasis in muscle and lipid metabolism. SCFAs produced by the gut microbiota activate AMP-activated protein kinase (AMPK) in muscle cells, thereby promoting muscle protein synthesis and energy metabolism. In contrast, endotoxins activate Toll-like receptors 4 and 5 in skeletal muscle, leading to nuclear factor κB (NF-κB) activation and subsequent inflammatory cytokine release, which contributes to muscle inflammation and atrophy (Lu et al., 2019). Gut microbial dysbiosis can disrupt muscle metabolism, thereby promoting the onset and progression of sarcopenia. In germ-free mouse models, the absence of gut microbiota resulted in reduced skeletal muscle mass due to muscle atrophy (Lahiri et al., 2019). Furthermore, gut microbial dysbiosis not only alters muscle protein synthesis and degradation but also impairs muscle mass by disrupting mitochondrial function and increasing oxidative stress. Clinically, alterations in the gut microbiota are closely associated with sarcopenia in older adults (Kang et al., 2021), and probiotic supplementation has been shown to improve muscle mass and function in patients with sarcopenia (Ren et al., 2025). The gut microbiota regulates nutrient metabolism, modulates protein synthesis and degradation, controls inflammatory responses, maintains mitochondrial function, and ultimately influences muscle metabolism and function. Overall, these mechanisms form the basis of the gut muscle axis concept and provide important theoretical support for understanding sarcopenia pathogenesis and developing novel therapeutic strategies.

#### 4.1.4 The influence of intestinal Flora on motor function

The gut microbiota exerts a significant influence on motor function. SCFAs play essential roles in energy supply, metabolic regulation, immune modulation, and maintenance of intestinal barrier integrity (Dziewiecka et al., 2022). Exercise enhances dietary fiber fermentation by the gut microbiota to produce acetate, propionate, and butyrate, thereby increasing SCFAs production and improving host metabolic health and exercise performance (Huang and Qi, 2024). Studies have shown that, compared with sedentary individuals, physically active individuals exhibit significantly higher fecal SCFAs levels. Studies of marathon runners have demonstrated that fecal propionate and butyrate levels increase significantly after the race (Li et al., 2018). Among SCFAs, butyrate serves as the primary energy source for colonic epithelial cells, promoting cellular proliferation and differentiation while enhancing intestinal barrier function. Acetate and propionate enter the circulation and participate in hepatic regeneration and regulation of lipid metabolism. Exercise also influences other metabolic pathways involving the gut microbiota (Liu and Qiu, 2023a). Amino acid and carbohydrate metabolic pathways within the gut microbiota of athletes exhibit higher activity. Exercise modulates the gut microbiota, influences enterohepatic bile acid circulation, alters secondary bile acid production, and in turn affects lipid metabolism and intestinal immunity.

### 4.2 The impact of exercise on intestinal Flora

**Figure 3:**
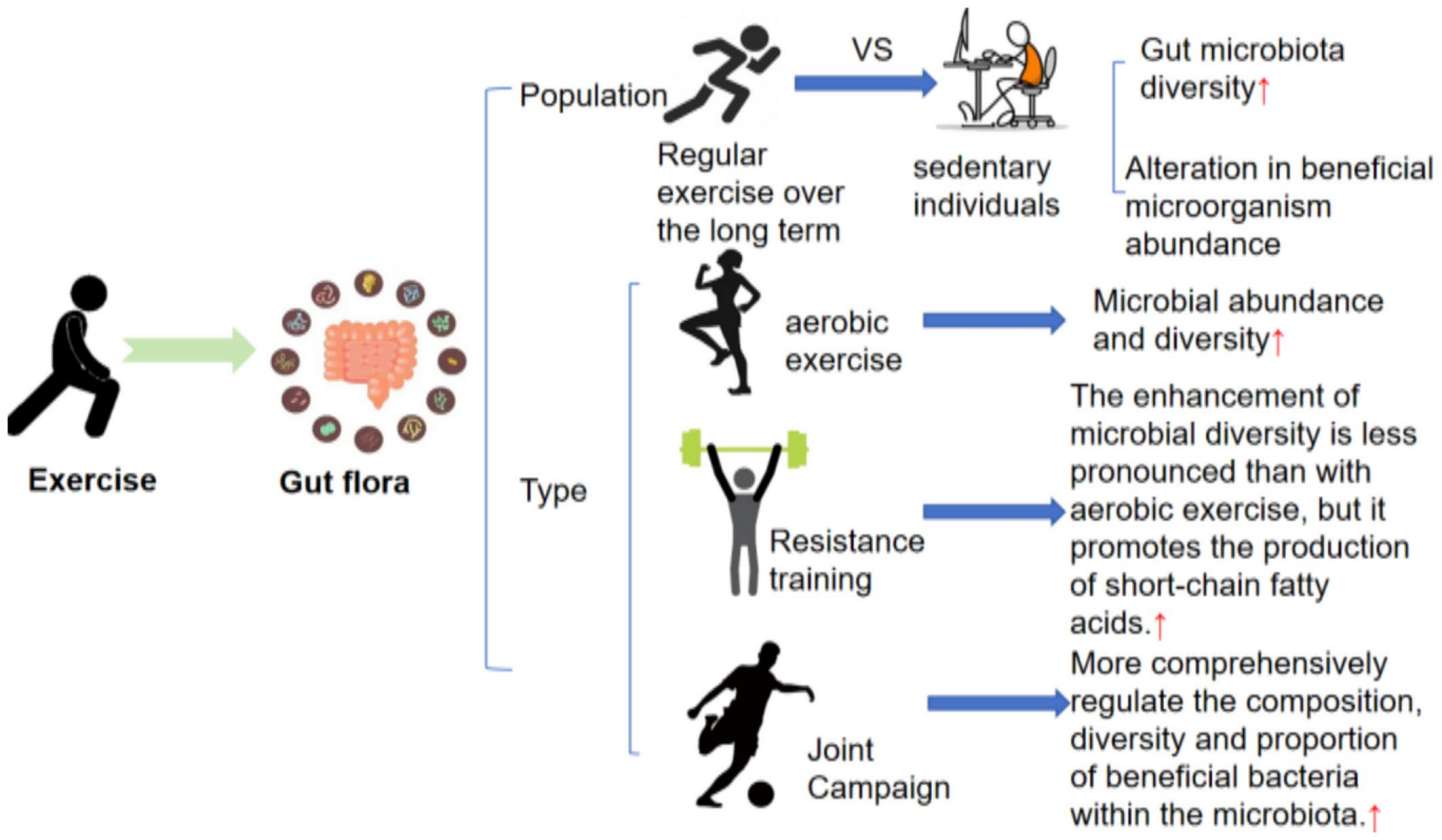
Illustrates the distinct effects of exercise modalities and intensities on the composition of the intestinal microbiota.

Different types and intensities of exercise exert distinct effects on the composition of the intestinal microbiota (Zhang et al., 2022) (Figure 3). A cross-sectional study examining different exercise intensities and durations demonstrated that, compared with sedentary individuals, long-term exercisers exhibited greater gut microbiota diversity, along with alterations in the relative abundance of beneficial bacteria (Wegierska et al., 2022). A randomized controlled trial involving professional football players and long-term inactive individuals demonstrated that the diversity of *Firmicutes* in athletes was significantly increased, accompanied by elevated relative abundances of *Clostridium, Vibrio*, *Helicobacter*, and *Faecalococcus. Clostridium* is a key butyrate-producing bacterium with anti-inflammatory properties, and an increase in its abundance contributes to the maintenance of a healthy intestinal microenvironment. Exercise intensity and duration also influence the gut microbiota. Most review studies have confirmed that moderate-intensity, long-term, and regular exercise is beneficial for the gut microbiota. However, prolonged excessive exercise and high-intensity endurance training may exert negative effects on the gut microbiota. Elite cyclists undergoing long-term, high-intensity training exhibit reduced gut microbiota stability and increased relative abundances of opportunistic pathogens, which are associated with impaired intestinal barrier function and an elevated risk of inflammation (Cataldi et al., 2022). With respect to exercise modality, aerobic and resistance training exert distinct effects on gut microbiota regulation. Aerobic exercise can significantly enhance gut microbiota richness and diversity, which are closely associated with improved metabolic function and strengthened intestinal barrier integrity. A 12-week low-intensity continuous aerobic exercise program in premenopausal women resulted in a significant increase in the abundance of *Akkermansia muciniphila* and *Prevotella copri* in the exercise group (Clark and Mach, 2016). Resistance training is less effective than aerobic exercise in enhancing bacterial diversity; however, it can promote the growth of SCFAs producing bacteria in the intestine (Bycura et al., 2021). Combined exercise programs incorporating both aerobic and resistance training can produce a more comprehensive gut microbiota profile and increase both the diversity and relative abundance of beneficial bacteria.

## 5 The relationship between intestinal flora and diseases

Research evidence suggests that both regular exercise and a healthy gut microbiota can independently exert positive preventive and therapeutic effects on a variety of diseases. However, most existing studies have concentrated on the combined impact of exercise and diet on the gut microbiota under pathological conditions. Systematic experimental data to support direct evidence and elucidate the specific mechanisms underlying the causal relationship between exercise and the re-regulation of disease progression through the gut microbiota remain insufficient. Therefore, this subsection aims to explore the potential mechanisms by which exercise prevents and treats diseases through the regulation of the gut microbiota and its metabolites. Recent studies have confirmed that exercise can regulate the structure of the intestinal microbiota and enhance intestinal barrier function via the gut-organ axis, playing a role in the prevention and treatment of chronic non-communicable and immune diseases through this pathway. This provides a novel theoretical basis and strategic framework for expanding the application of exercise in the prevention and treatment of other diseases. This article considers the gut microbiota as the core mediating variable and systematically reviews, guided by organ systems, the physiological and molecular mechanisms through which exercise interventions influence disease prevention and treatment by altering the composition of the gut microbiota and its metabolites, aiming to provide theoretical insights for subsequent research in this field.

### 5.1 Association between gut microbiota and trauma-related diseases

**Figure 4:**
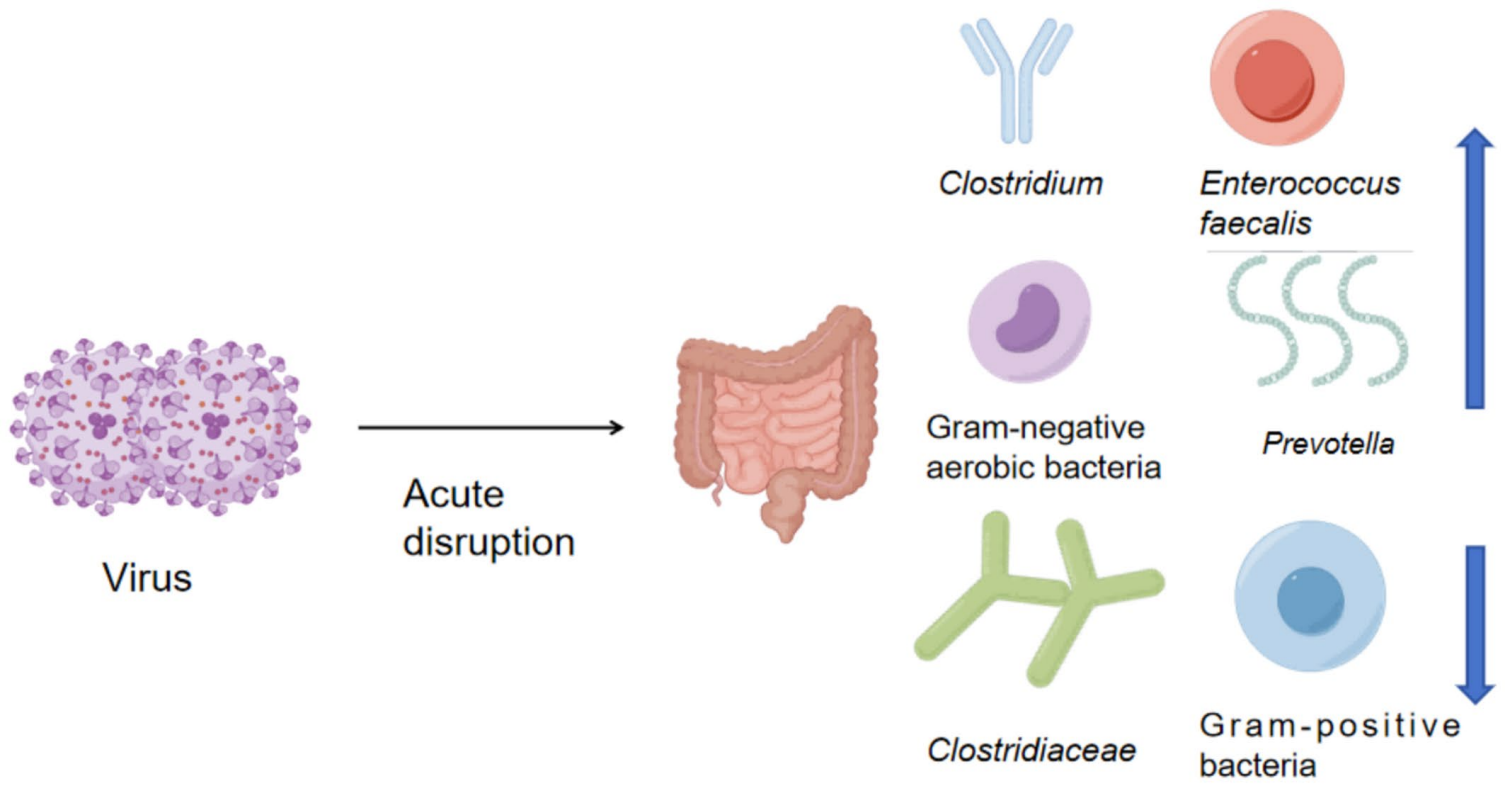
Changes in gut microbiota associated with traumatic diseases.

Stress caused by acute diseases or injuries can significantly disrupt the balance of the gut microbiota, and this disruption can increase susceptibility to inflammation or infection, thereby affecting functional changes in the body’s inflammatory response (Figure 4). Current research has established the connection between external trauma and the gut microbiota, initially observed in animal models. In human clinical trials, excessive proliferation of *Gram-negative pathogenic bacteria* was detected within 24 to 72 h of illness onset in patients. During the early post-trauma period (within 72 h), alpha diversity of the gut microbiota decreased, while beta diversity also showed a decline (Munley et al., 2023). A significant increase in the abundance of *Clostridium* and *Enterococcus faecalis* (Howard et al., 2017) is associated with inflammatory manifestations in the body, though large-scale evidence-based clinical studies are still lacking. Early research primarily focused on descriptive studies of gut microbiota changes induced by trauma, particularly analyzing bacterial translocation manifestations. Current research focuses on the molecular mechanisms linking gut microbiota and external trauma, with the aim of repairing intestinal barrier function and utilizing probiotic interventions to alter the composition and function of the gut microbiota. In 2001, it was first reported that in a rat model of hemorrhagic shock, intestinal barrier damage occurred alongside bacterial translocation. With the development of burn, traumatic brain injury, and spinal cord injury models, researchers found that these acute injuries could induce significant intestinal microbiota imbalances (Xu et al., 2023). In 2016, human clinical observations detected excessive proliferation of *Gram-negative pathogenic* bacteria in blood and fecal samples from external trauma patients, suggesting a possible association between bacterial translocation and systemic inflammation. Burn patients are characterized by high infection rates, making the clinical research application particularly valuable. After burns, overall intestinal diversity decreases, intestinal permeability increases, *Gram-negative aerobic bacteria* overgrow, and the relative abundance of *Enterobacteriaceae* increases (Earley et al., 2015). The research literature on traumatic brain injury patients is relatively abundant, accounting for approximately 20% of external trauma cases. Patients with traumatic brain injury exhibit reduced gut microbiota diversity, with a significant decrease in *Clostridium* and Gram-positive bacteria, while the abundance of *Prevotella* and *Rikenellaceae* increases. In conclusion, post-trauma changes in the host’s gut microbiota are primarily characterized by a reduction in beneficial bacteria, an increase in pathogens, and a decrease in microbial diversity.

Exercise can reshape the gut microbiota and improve inflammatory manifestations in traumatic diseases. For example, 8 weeks of treadmill walking (30 min per day, 5 days a week) increases intestinal metabolite levels in a rat model of traumatic osteoarthritis, specifically increasing the abundance of *Fusobacteria* at the phylum level. At the genus level, the abundances of *Lactobacillus, Turicibacter*, *Adlercreutzia*, and *Cetobacterium* increased, while the increase in *Adlercreutzia* and *Cetobacterium* attenuated the exercise response (Hao et al., 2022). The research suggests that changes in the gut microbiota may reduce the expression of inflammatory factors in serum and improve the physiological processes associated with trauma-induced inflammation. Eight weeks of aerobic exercise can regulate gut microbiota composition, increase SCFAs levels, enhance Pitpnc1 protein expression, maintain mitochondrial fusion and division, and improve injury outcomes in mouse models of traumatic brain injury (Sun, 2023). Currently, there are relatively few animal or human studies on the effects of different exercises on trauma disease manifestations, and comprehensive experimental data are still lacking. Currently, most research on exercise regulation of gut microbiota to improve external trauma disease manifestations is focused on verifying aerobic exercise interventions in animal models, with limited human experimental data.

### 5.2 Association between gut microbiota and skin diseases

**Figure 5:**
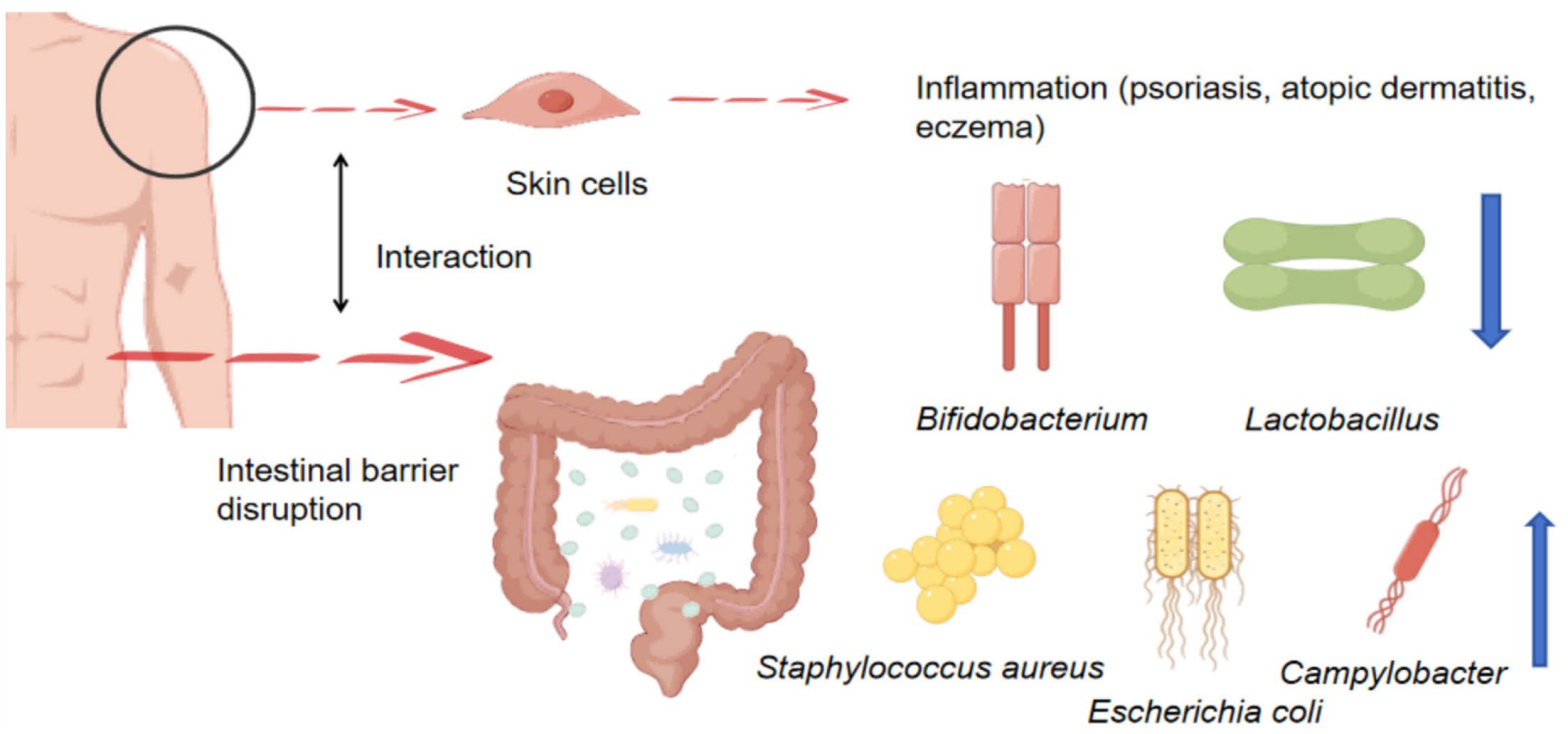
Changes in gut microbiota associated with skin diseases.

The changes in the gut microbiota associated with skin diseases are mainly reflected in a decrease in the relative abundance of beneficial bacteria and an increase in harmful bacteria (Figure 5). The primary pathogenic mechanism involves increased inflammatory factors that damage the skin barrier and trigger inflammatory responses. The association between skin diseases and the gut microbiota has become a research focus in recent years, primarily influencing intestinal and skin physiology through the bidirectional effects of the gut-skin axis. Current research on the association between skin diseases and the gut microbiota is characterized by short-term and small-scale studies, and there is a lack of large-scale, double-blind randomized controlled trials to verify the reversibility of this causal relationship. Due to the complexity of disease pathogenesis, both animal models and *in vitro* studies lack mechanistic investigations into how skin diseases regulate the gut microbiota to influence skin inflammation via the gut-skin axis. Current understanding of skin disease pathogenesis is largely based on descriptive studies of acne, atopic dermatitis, and psoriasis. Gut microbiota diversity in patients with psoriasis is significantly reduced, with decreased abundances of *Gram-positive anaerobic bacteria*, *Bifidobacterium*, and *Lactobacillus* species, while the abundance of pathogenic bacteria such as *Escherichia coli*, *Campylobacter*, and *Staphylococcus aureus* is increased. In contrast, patients with atopic dermatitis do not exhibit a significant reduction in gut microbiota diversity but show decreased abundances of *Propionibacterium acnes* and *Burkholderia*, along with increased abundances of *Clostridium* and *Neisseria* (Olejniczak-Staruch et al., 2021). *Patients with* eczema exhibit significantly reduced gut microbiota diversity, with decreased abundances of *Bifidobacterium* and *Lactobacillus* species and increased abundances of *Staphylococcus aureus* and *Candida albicans* (Reali et al., 2024). In conclusion, individuals with skin diseases exhibit significantly reduced gut microbiota diversity, accompanied by increased pathogenic bacteria and decreased beneficial bacteria.

Current research has confirmed that gut microbiota–targeted microecological interventions, including probiotics, prebiotics, synbiotics, and fecal microbiota transplantation, can be applied in the treatment of skin diseases. Fecal microbiota transplantation can restore gut microbiota composition to pre-disease levels in mouse models of specific dermatitis, increase intestinal short-chain fatty acid levels, and restore Th1/Th2 balance (Kim et al., 2021). Long-term oral administration of *Blautia* and *Bifidobacterium* can increase short-chain fatty acid concentrations in the gut microbiota and alleviate psoriasis symptoms in some patients (Liu et al., 2024). Low- and moderate-intensity exercise can regulate short-chain fatty acid levels, reduce the *Firmicutes/Bacteroidetes* ratio, alleviate intestinal inflammation, enhance intestinal barrier function (Secchiero et al., 2024), and maintain a balanced Th1/Th2 ratio (Supriya et al., 2021). Current research has confirmed an association between exercise, gut microbiota modulation, and the treatment of skin diseases. However, large-scale experimental data confirming that exercise induces improvements in gut microbiota composition and its metabolites in skin diseases are still lacking. At present, most studies have identified key bacterial genera associated with skin disease improvement, and low- and moderate-intensity exercise can induce changes in these genera. However, the mechanisms by which exercise-induced metabolites regulate skin immune responses have not been fully elucidated. In the future, extensive human experimental data will be required to verify the relationships among exercise, gut microbiota, and skin diseases.

### 5.3 The association between gut microbiota and ocular diseases

**Figure 6:**
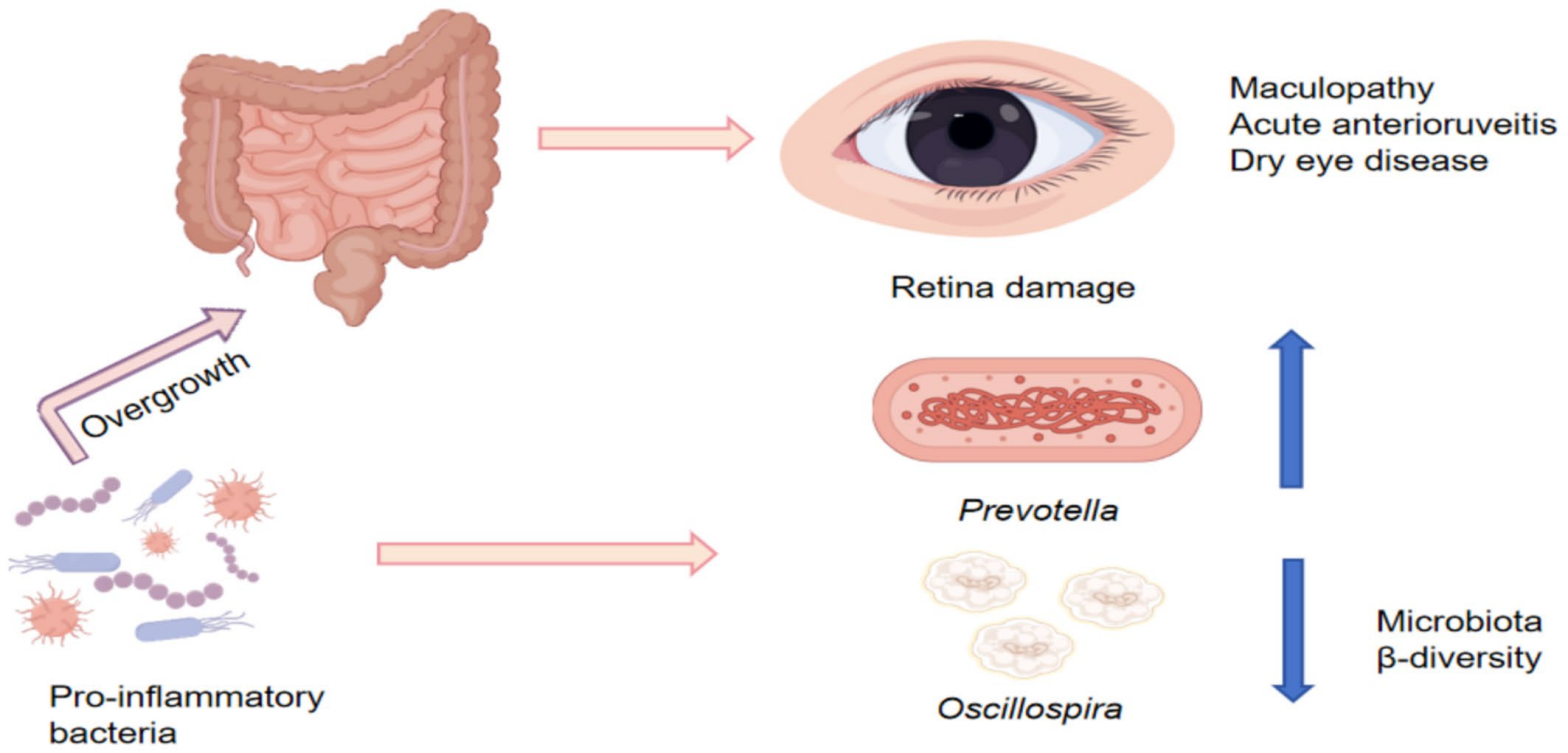
Alterations in gut microbiota associated with eye diseases.

The relationship between the gut microbiota and ocular diseases is primarily mediated through the gut-eye axis. Gut microbiota homeostasis affects the proliferation and morphological characteristics of ocular surface bacteria (Figure 6). In 2015, early animal experiments demonstrated that antibiotic treatment could inhibit experimental uveitis, confirming a functional link between the intestine and the eye. Subsequent research expanded from single-disease pathogenesis to a broader framework encompassing multiple ophthalmic diseases and shifted from correlation analyses to functional and mechanistic investigations. However, at the human level, current evidence still relies largely on associative data, and the therapeutic mechanisms remain unclear. Current research indicates that patients with ocular diseases exhibit gut microbiota dysbiosis, in which excessive proliferation of pro-inflammatory bacteria disrupts the intestinal barrier and induces metabolic endotoxemia, systemic inflammation, and retinal damage (Zysset-Burri et al., 2023). A randomized controlled trial showed that among 57 patients with age-related macular degeneration, *Gram-negative bacteria* increased, whereas the abundance of *Vibrio* species decreased. An animal study showed that transgenic rats carrying the human HLA-B27 gene exhibited reduced relative abundances of *Bacteroides* and *Bacteroidaceae*, along with an increased abundance of Prevotella (Lin et al., 2014). Clinical studies have demonstrated increased abundances of pro-inflammatory and pathogenic bacteria (*Veillonella*, *Prevotella*, and *Streptococcus*) in patients with acute anterior uveitis, accompanied by reduced abundances of protective bacteria such as *Roseburia*, *Faecalibacterium*, *Ruminococcus*, and *Helicobacter* (Kalyana Chakravarthy et al., 2018; Huang et al., 2018). Animal studies have shown that antibiotic-treated dry eye mice exhibit reduced abundances of *Clostridium* and increased growth of pro-inflammatory and pathogenic bacteria (*Enterobacter*, *Pseudomonas*, and *Escherichia/Shigella*), leading to worsened disease symptoms and reduced goblet cell density, a major source of tear mucins (de Paiva et al., 2016). In conclusion, alterations in gut microbiota *β* diversity and enrichment of *Proteobacteria*, *Prevotella*, *Spirochaetes*, and anaerobic amoebae are positively correlated with ocular disease severity (Trujillo-Vargas et al., 2020); however, direct evidence linking gut microbiota–derived TMAO and choline metabolic pathways to ocular disease regulation remains limited. At present, there is a lack of randomized controlled trials directly examining the association among exercise, gut microbiota, and ocular diseases. Nevertheless, aerobic exercise has been shown to increase the abundance of butyrate-producing bacteria and elevate circulating short-chain fatty acid concentrations (Davis et al., 2021). Butyrate can inhibit histone deacetylases and activate G protein–coupled receptors GPR41, GPR43, and GPR109A, thereby exerting antioxidant and anti-inflammatory effects (Martin-Gallausiaux et al., 2021). It is therefore speculated that exercise-induced gut microbiota alterations may participate in inhibiting the progression of age-related macular degeneration. A*kkermansia* has been identified as a protective factor in visual function. Visual decline may contribute to the co-occurrence of multiple ocular diseases, and reduced abundance of *Akkermansia* is associated with an increased risk of myopia, highlighting the gut microbiota as a potential pathway for vision improvement. Exercise can promote the production of indole-3-acetic acid, a gut microbiota–derived metabolite, and increase expression of the transcription factor SP1, thereby enhancing COL1A1 expression and maintaining scleral structural integrity (Li et al., 2024). These findings provide a novel approach for the prevention and treatment of ocular diseases and visual impairment. By promoting intestinal peristalsis, exercise may enhance gut microbiota health and increase the relative abundance of *Akkermansia*, suggesting that exercise-mediated regulation of the gut microbiota may confer potential benefits in improving ocular diseases.

### 5.4 The association between gut microbiota and liver diseases

**Figure 7:**
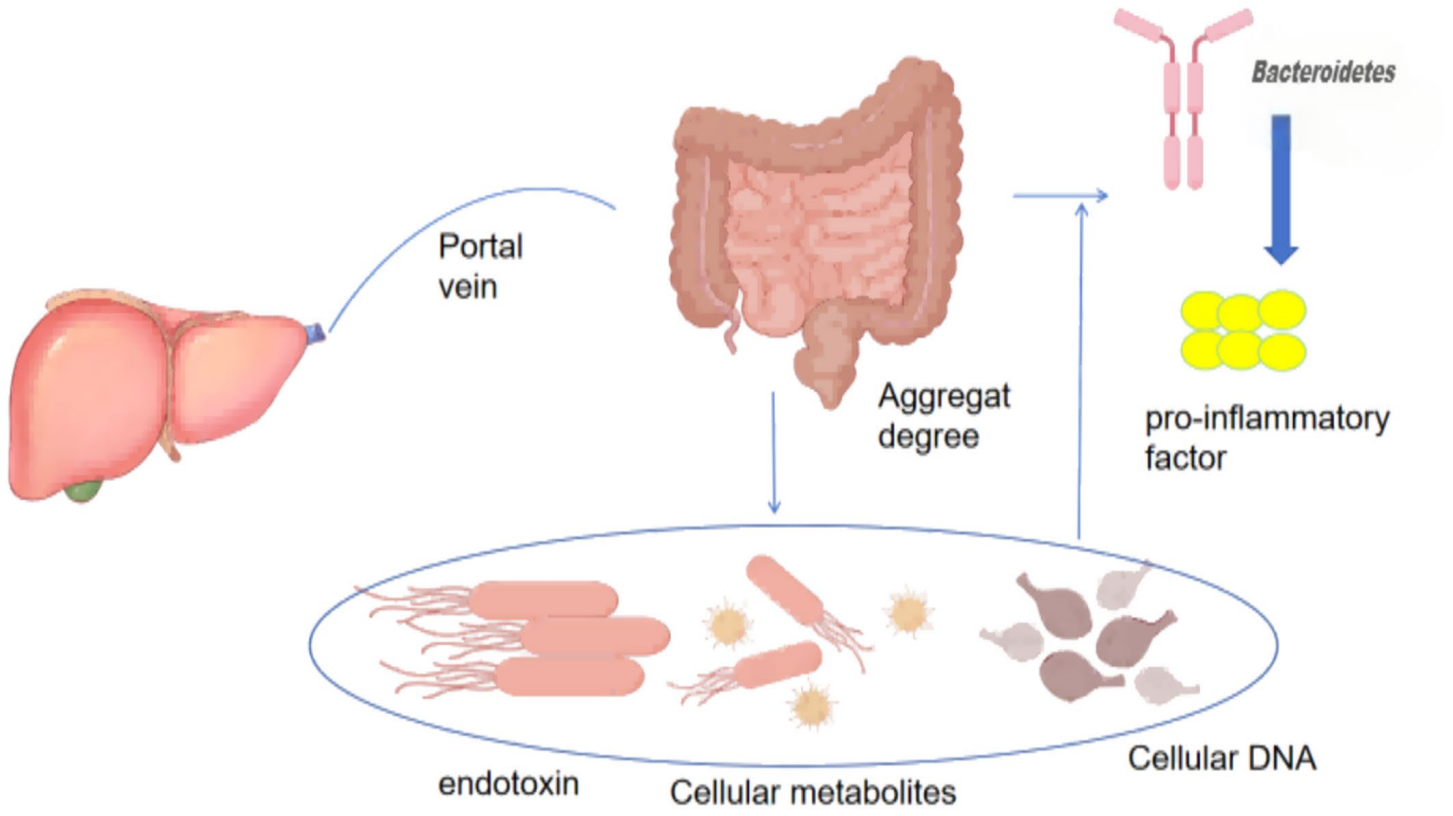
Alterations in gut microbiota associated with liver diseases.

The primary pathway for intestinal nutrient absorption is the hepatic portal vein. Disruption of intestinal barrier integrity can impair hepatic filtration, thereby promoting the systemic accumulation of gut microbiota–derived products, including endotoxins, bacterial metabolites such as peptidoglycan, and bacterial DNA (Haque and Barritt, 2016). This pathway mediates the influence of the gut microbiota on normal liver function. Endotoxins bind to Toll-like receptor 4 on hepatic cells and promote liver fibrosis (Figure 7). At low concentrations, endotoxins activate Kupffer cells to clear hepatic pathogens, triggering inflammatory cascades and cytokine release, which can lead to liver injury and increase the risk of cirrhosis even in healthy individuals (Chassaing et al., 2014).

In addition, the gut microbiota regulates bile acid metabolism by activating or inhibiting farnesoid X receptor(FXR) and TGR5, thereby influencing hepatobiliary metabolism (Wang et al., 2018). The gut microbiota also influences cholesterol metabolism through trimethylamine production and subsequent trimethylamine N-oxide generation, accelerating hepatic lipid deposition. Most metabolic pathways associated with the gut–liver axis have been validated in animal models. However, findings from animal models cannot be directly extrapolated to humans. Because experimental animals are typically treated with antibiotics, it remains difficult to determine whether observed microbiota alterations are causal or consequential. Most current human clinical studies focus on the LPS–TLR4 pathway, the bile acid–FXR/TGR5 axis, SCFAs, inflamma some-mediated pyroptosis, and immunomodulatory pathways. However, large-scale and standardized intervention trials are still lacking to confirm the regulatory effects of these pathways on the gut microbiota.

Pathological evidence indicates that patients with non-alcoholic fatty liver disease exhibit reduced abundance of *Bacteroidetes* (Mouzaki et al., 2013) and relatively low enrichment of *Ruminococcus* (Raman et al., 2013). In summary, gut–liver interactions are primarily mediated through hepatic filtration pathways, influencing liver disease prevalence by modulating endotoxin binding to Toll-like receptor 4, reducing pro-inflammatory factor expression, and enhancing intestinal barrier homeostasis. Exercise can regulate the gut–liver axis to prevent and treat non-alcoholic fatty liver disease by modulating gut microbiota–derived metabolites, including bile acids, butyrate, and ethanol (Table 1). Six- and 12-week exercise interventions increase expression of the cholesterol 27α-hydroxylase gene in mouse models (Pinto et al., 2018), accelerating hepatic cholesterol uptake, enhancing conversion of cholesterol to bile acids, reducing biliary cholesterol saturation, and promoting bile acid synthesis (Wilund et al., 2008). Moderate-intensity aerobic exercise can normalize gut microbiota structure in high-fat diet–fed mice, increase the abundance of *Actinobacteria*, reduce *Bacteroidetes* abundance (Wan, 2017), and elevate short-chain fatty acid levels. These changes reduce hepatic lipid droplets, significantly decrease inflammatory markers TLR4 and TNF-*α*, and restore intestinal barrier function (Zhang and Sang, 2022; Carbajo-Pescador et al., 2019). In patients with non-alcoholic fatty liver disease, aerobic exercise performed for 60 min three times weekly over twelve weeks increases the gut microbiota Shannon index and elevates the relative abundance of *Faecalibacterium prausnitzii* and *Bifidobacterium*, accompanied by reduced hepatic fat content and decreased alanine aminotransferase and aspartate aminotransferase levels. Aerobic exercise combined with dietary intervention further enhances intestinal microecosystem stability, thereby improving hepatic lipid accumulation. Moreover, pre-intervention gut microbiota network characteristics can identify key bacterial changes associated with liver-related diseases and help explain individual responses to exercise and dietary interventions (Cheng et al., 2022). Existing studies indicate that exercise-induced modulation of the gut microbiota contributes to improved outcomes in liver-related diseases. However, extensive animal and human studies are still required to clarify the underlying mechanisms, establish causal relationships, and elucidate interactions among exercise, diet, pharmacological interventions, and liver disease progression.

**Table 1:** Mechanistic pathways by which exercise modulates the gut microbiota in liver related diseases.

Disease type	Sports	Before intervention	After the intervention	Possible mechanisms of action
Nonalcoholic fatty liver disease	Aerobic exercise	Hepatic steatosis associated with severe insulin resistance and intestinal dysbiosis, which promotes the translocation of endotoxins into the bloodstream.	Improvement in Liver FunctionParameters, Reduction in Liver Enzyme Levels, Enhanced Insulin Sensitivity, and Restoration of Gut MicrobiotaBalance	Aerobic exercise increases energy expenditure and fatty acid oxidation, thereby reducing hepatic fat deposition and improving enterohepatic circulation. These effects decrease endotoxin translocation, promote the proliferation of *Akkermansia*, and enhance the production of bacterial, which strengthen intestinal barrier function and regulate hepatic glycolipid metabolism (Fu et al., 2023).
	High intensity interval training			High-intensity interval training activates AMPK activated protein kinase signaling and improves mitochondrial function, thereby enhancing systemic insulin sensitivity. This metabolic improvement reduces free fatty acid influx into the liver, modulates the metabolic product profile of the gut microbiota, and ultimately inhibits hepatic inflammatory and fibrotic pathways (Oh et al., 2017).
	Aerobic combined resistance training		Concurrent reductions in hepatic steatosis and fibrosis related markers	Joint training synergistically improves insulin sensitivity and muscle mass, thereby correcting metabolic abnormalities at the systemic level. These adaptations promote the establishment of an anti-inflammatory gut microbiota, whose metabolites influence hepatocyte metabolism and inflammatory responses via the portal venous system (Zhong et al., 2022).

### 5.5 The association between gut microbiota and cardiovascular diseases

**Figure 8:**
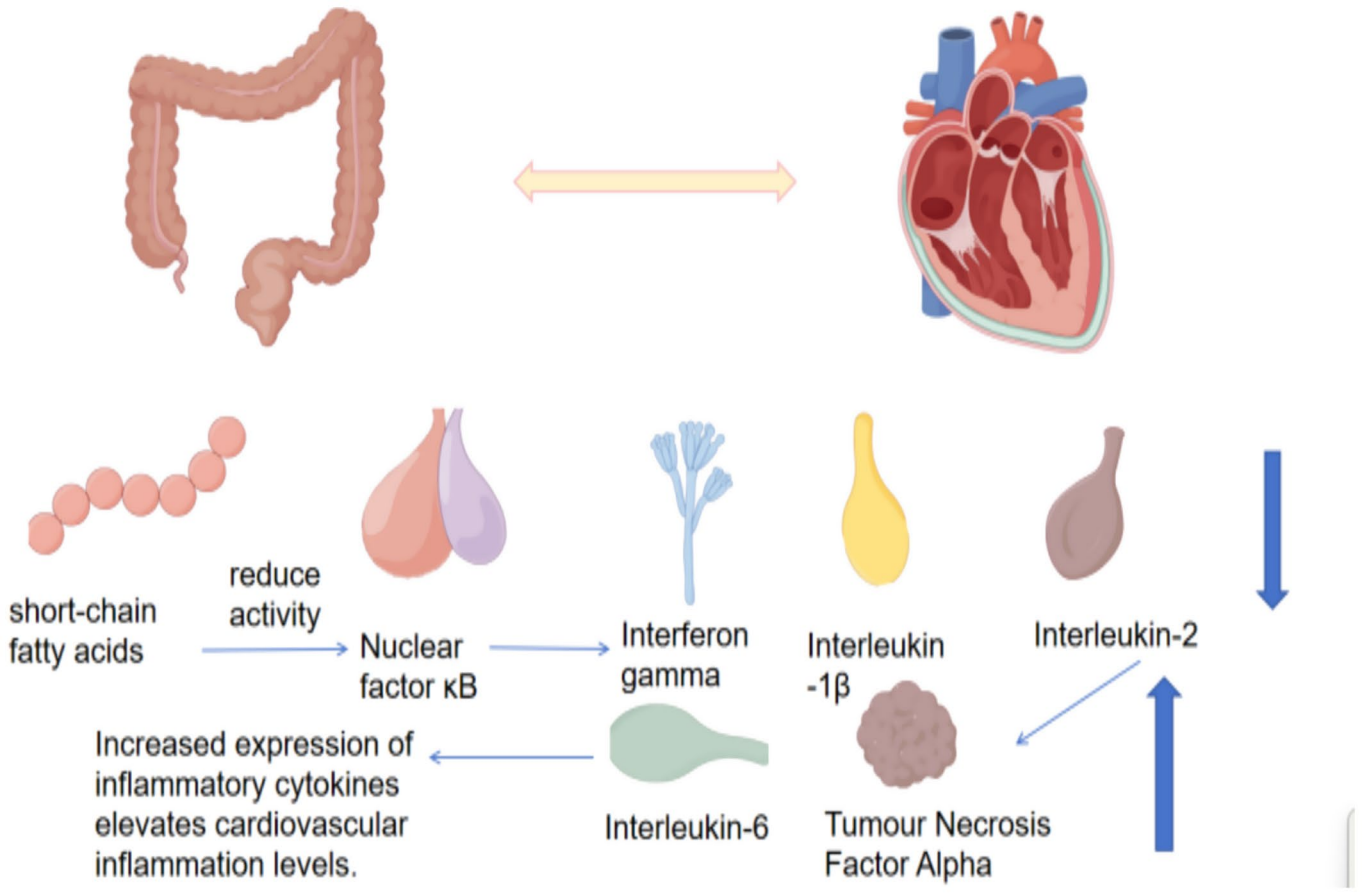
Alterations in the intestinal microbiota associated with cardiovascular diseases.

The regulation of cardiovascular diseases by the gut microbiota primarily depends on bidirectional interactions between the gut microbiota and the heart (Figure 8). Gut microbiota dysbiosis and the production of gut microbiota–derived metabolites can affect the expression of cardiovascular inflammatory factors, leading to increased systemic inflammation. SCFAs can inhibit NF-κB activity in immune cells and reduce the expression of pro-inflammatory cytokines, including interferon-*γ*, interleukin-1β, and interleukin-2 (Zou et al., 2022), whereas endotoxins upregulate inflammation-related genes and significantly increase plasma concentrations of tumor necrosis factor-*α* and interleukin-6 (Makrecka-Kuka et al., 2020). Early epidemiological association studies reported that gut microbiota composition in patients with coronary heart disease and hypertension differs from that of healthy individuals. Subsequent animal experiments confirmed the role of the gut microbiota and its metabolites in cardiovascular diseases. Human studies primarily focused on microbiota analyses in patient cohorts, whereas conclusions from long-term follow-up studies remained inconsistent. Whether gut microbiota alterations precede cardiovascular disease onset or occur as a consequence of disease progression remains unclear. Current research has confirmed gut microbiota alterations in both animal and human disease models. In apolipoprotein E knockout mice, plasma lipopolysaccharide levels were significantly reduced in microbiota-modulated treatment groups, along with lower levels of pro-atherosclerotic inflammatory mediators, including interleukin-2 and interleukin-4, compared with untreated controls (Yoshida et al., 2018). Multiple cross-sectional and cohort studies have shown that the abundance of *Enterobacteriaceae*, including *Escherichia coli*, *Klebsiella*, and *Enterobacter*, is higher in fecal samples from patients with ischemic heart disease than in healthy controls. The relative abundance of oral bacteria such as *Streptococcus* and *Lactobacillus salivarius* was also increased (Jie et al., 2017). The gut microbiota may contribute to the development and progression of hypertension by influencing sodium absorption and short-chain fatty acid metabolism (Katsimichas et al., 2019). Trimethylamine N-oxide derived from the gut microbiota inhibits carnitine synthesis and fatty acid oxidation, thereby promoting cardiac hypertrophy. Prospective heart failure cohort studies have shown that TMAO levels increase in parallel with cardiac mortality and transplant risk (Zhao et al., 2022). In conclusion, the association between the gut microbiota and cardiovascular diseases is primarily mediated by gut microbiota–derived metabolites, which indirectly affect blood circulation and ultimately influence cardiac function and cardiovascular health.

Exercise can facilitate the systemic circulation of gut microbiota–derived metabolites, thereby improving cardiovascular disease–related symptoms (Table 2). Previous studies have shown that gut microbiota composition is altered in patients with cardiovascular diseases. Favorable changes in gut microbiota composition have been observed in exercise-trained mice, and human studies indicate that athletes exhibit greater gut microbiota richness and diversity. The gut microbiota exhibits modifiable characteristics during therapeutic interventions; therefore, targeting gut microbiota regulation provides a novel approach for combating chronic diseases. A four-week treadmill exercise intervention increased the relative abundance of *Butyricimonas* and *Akkermansia*, reduced cardiac output and stroke volume in myocardial infarction mice, and decreased serum inflammatory marker expression. Key operational taxonomic units included *Bacteroidetes*, *Barnesiella*, *Helicobacter*, *Parabacteroides*, *Porphyromonadaceae*, and *Ruminococcaceae*. Alterations in Ureaplasma abundance were associated with exercise and cardiovascular function (Zhang et al., 2017). An eight-week treadmill exercise experiment demonstrated that *Alistipes* and *Ruminococcus* were positively correlated with cardiac ejection fraction and fractional shortening, whereas *Lachnospiraceae* UCG-001 showed negative correlations. These microbiota alterations induced significant changes in 3-HPA and 4-HBA, activating NRF2 signaling to reduce OGD/R-induced cardiomyocyte apoptosis and improve cardiac dysfunction in myocardial infarction (Zhou et al., 2022). Based on existing evidence, exercise-induced gut microbiota modulation alters metabolite profiles, providing a microbiota–metabolism–cardiac axis perspective for cardiovascular disease prevention and treatment. Future studies should further explore the molecular mechanisms by which different exercise regimens regulate the gut microbiota and influence cardiovascular metabolic pathways. This will provide a theoretical basis for developing cardiovascular exercise rehabilitation strategies targeting gut microbiota.

**Table 2:** Mechanistic pathways by which exercise modulates the gut microbiota in cardiovascular related diseases.

Disease type	Sports	Before intervention	After the intervention	Possible mechanisms of action
Atherosclerosis	Moderate-intensity aerobic exercise	Impaired vascular endothelial function and gut microbiota dysbiosis may contribute to increased oxidative stress and atherosclerotic plaque formation.	An optimized blood lipid profile, improved vascular endothelial function, reduced inflammatory markers, decreased arterial stiffness, increased gut microbiota diversity, and enrichment of beneficial bacterial taxa were observed.	Aerobic exercise enhances nitric oxide bioavailability and improves endothelial function, thereby modulating gut microbiota composition and increasing the abundance of short chain fatty acid producing bacteria. Microbiota derived metabolites, including butyrate, circulate systemically to inhibit vascular inflammation and reduce the accumulation of oxidized low density lipoprotein (LDL) (Zhong et al., 2022).
	Moderate resistance training			Resistance training enhances skeletal muscle glucose uptake and lipid oxidation, thereby improving systemic metabolic status. These metabolic adaptations reduce metabolic endotoxemia and promote favorable alterations in gut microbiota structure, while microbiota derived metabolites contribute to the regulation of cholesterol metabolism and the bile acid cycle (de Luca et al., 2024).
	Aerobic and Resistance Combined Training			Concomitant training leads to improvements in metabolism, inflammation, and vascular function, resulting in the comprehensive optimization offrom the microbiota may be regulated, influencing plaque stability (de Luca et al., 2024).
Hypertension	Aerobic exercise	Increased sympathetic nervous system activity, elevated vascular resistance, and gut microbiota dysbiosis may contribute to dysregulation of sodium metabolism and vascular tone.	Decreased resting blood pressure, improved heart rate variability, and enhanced vasodilatory function were observed.	Aerobic exercise modulates autonomic nervous system balance by reducing sympathetic activity and enhancing parasympathetic tone, thereby improving vascular endothelial function. These effects contribute to favorable modulation of the gut microbiota, leading to increased production of. Microbiota derived activate vascular G protein coupled receptors, promoting vasodilation and regulation of the renin angiotensin system (Avery et al., 2021).
	Yoga/Breathing Training		Decreased blood pressure variability was observed.	Psychosomatic interventions reduce sympathetic nervous system activity and cortisol levels, thereby alleviating stress induced increases in intestinal permeability. These effects support the maintenance of microbial homeostasis, while microbiota derived metabolites participate in the regulation of central and peripheral blood pressure control circuits (Wu et al., 2023).

### 5.6 Association between gut microbiota and lung diseases

**Figure 9:**
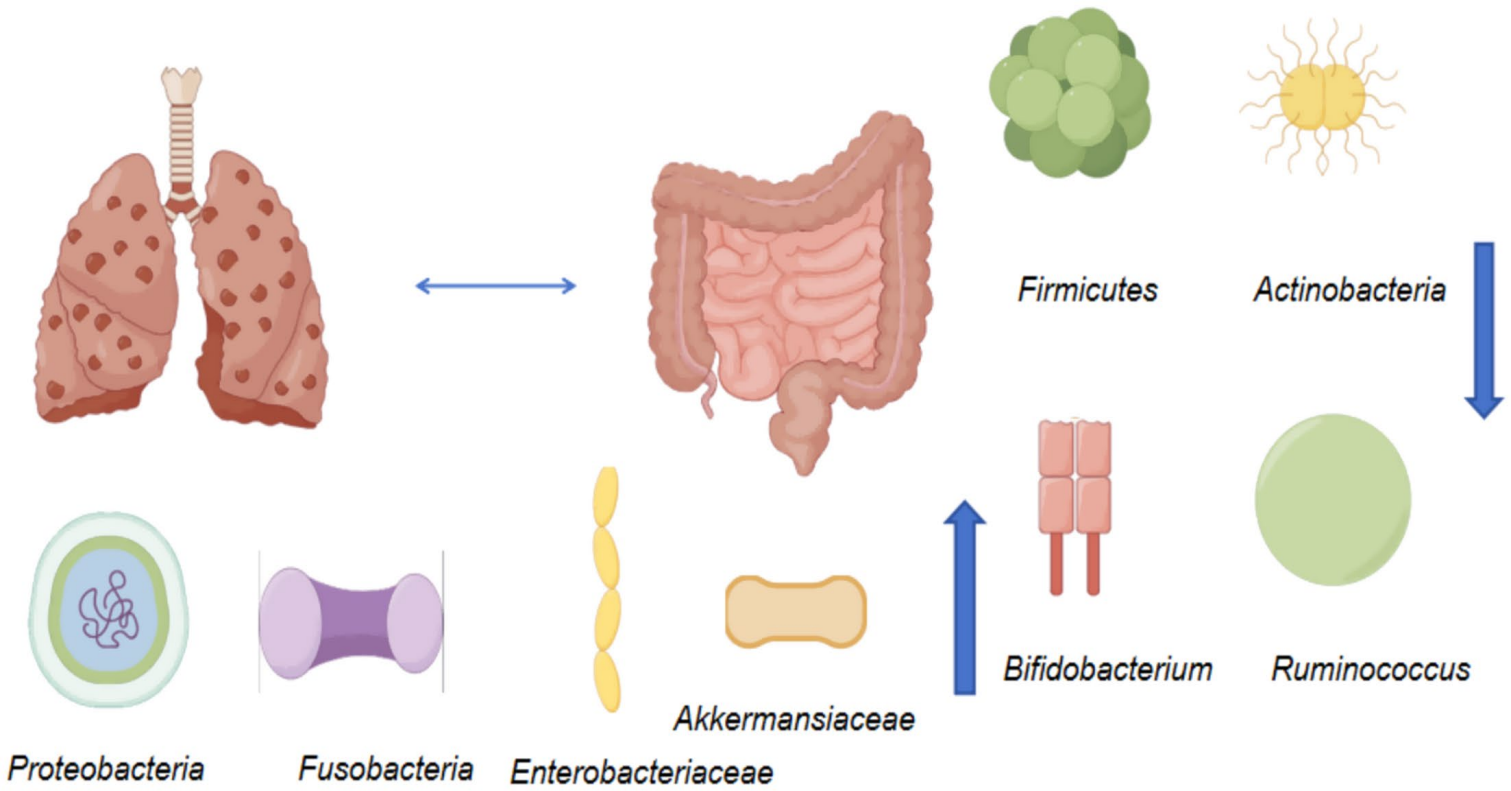
Alterations in gut microbiota associated with lung diseases.

**Figure 10:**
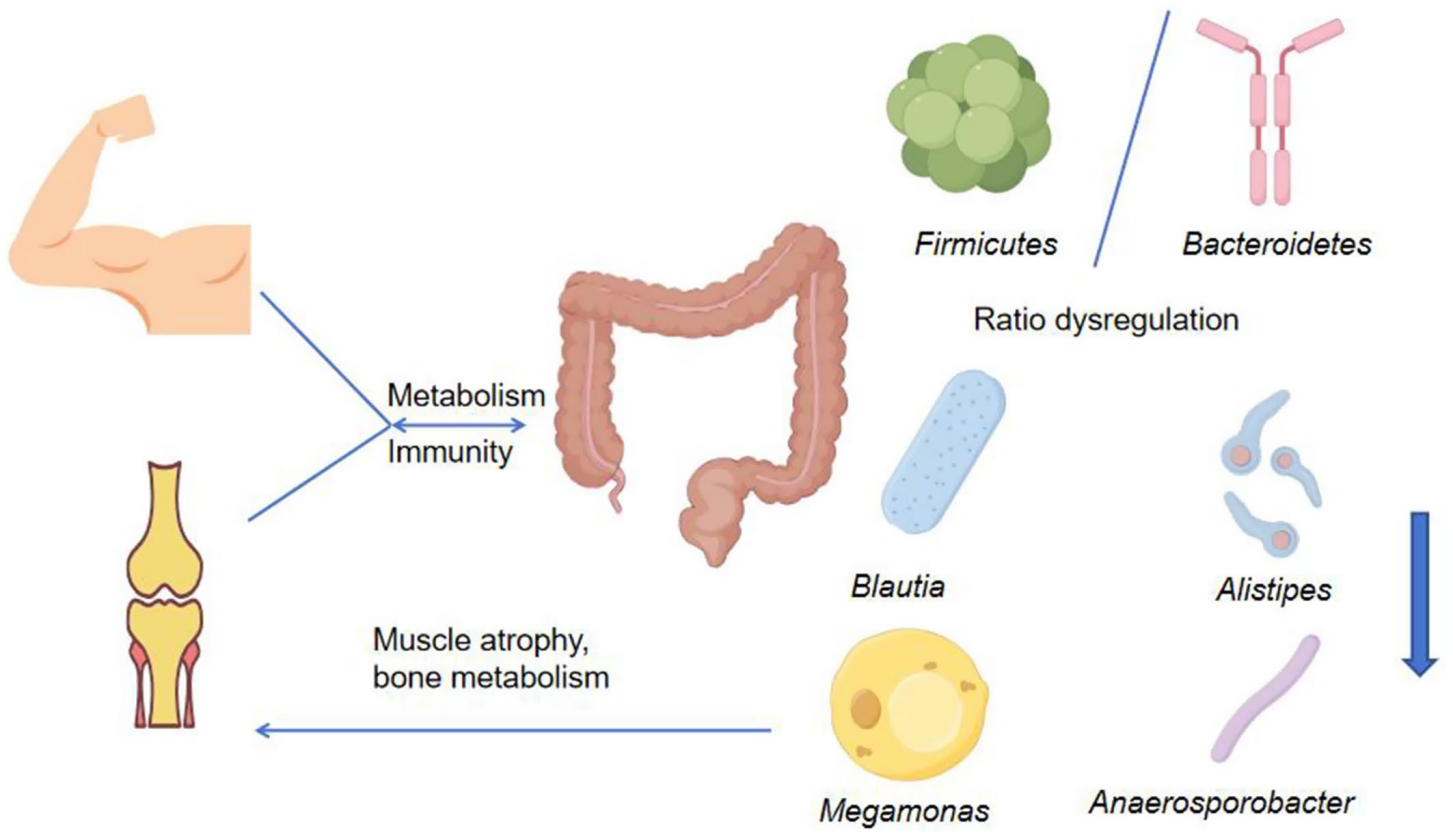
Changes in intestinal flora affected by skeletal muscle diseases in review.

The impact of the gut microbiota on the onset of lung diseases is characterized by reduced gut microbial diversity, a decrease in beneficial commensals, and an increase in opportunistic pathogens (Figure 9). In 2010, 16S rRNA high-throughput sequencing first enabled the description of the composition of both lung and intestinal microbiota. Initially, this research primarily revealed alterations in the intestinal microbiota induced by lung diseases, confirming the existence of the gut-lung axis. Animal models further corroborated that gut microbiota dysbiosis exacerbates lung inflammation. Human cross-sectional studies have confirmed a reduction in gut microbial diversity among patients with asthma and chronic obstructive pulmonary disease. However, confounding variables such as smoking, excessive alcohol consumption, and diet contributing to individual differences in lung patients must be considered, alongside a notable absence of long-term follow-up investigations. Subsequent research has shifted its focus to elucidating the mechanistic pathways of the gut-lung axis. The association between the gut microbiota and the lungs is bidirectionally mediated through immune modulation, metabolic signaling, and maintenance of barrier function. Chronic obstructive pulmonary disease is associated with a significant decrease in the proportion of *Firmicutes* and *Actinomycetes*, alongside a marked reduction in the abundance of beneficial bacteria such as *Bifidobacterium* and *Ruminococcus*. Conversely, the proportions of *Proteobacteria* and *Clostridium* demonstrated a significant increase (Li et al., 2021). Opportunistic pathogens such as *Enterobacteriaceae* and *Akkermansia* also exhibited an increased proportion (Figure 10). Asthma patients exhibit gut dysbiosis, characterized by an altered *Bacteroidetes/Firmicutes* ratio, a significant increase in *Proteobacteria*, a decreased abundance of *Bifidobacterium* and *Ruminococcus*, and an increased abundance of *Haemophilus* (Hilty et al., 2010). Patients with severe pneumonia demonstrate a significant reduction in gut microbial diversity, accompanied by a decrease in the proportions of *Firmicutes* and *Bacteroidetes*, and a substantial reduction in beneficial bacteria such as *Bifidobacterium*, *Ruminococcus*, and *Pseudomonas*. Conversely, the proportion of *Actinomycetes* and mutuotrophs increased, while that of opportunistic pathogenic bacteria such as *Escherichia* and *Clostridium* also rose (Yu et al., 2023). In lung cancer patients, the gut microbiota exhibits an imbalance in the *Bacteroidetes/Firmicutes* ratio, and the abundance of *Prevotella* is significantly reduced. The proportion of *Proteobacteria* and *Actinomycetes* increased significantly, as did the abundance of bacteria such as *Streptococcus* and *Streptococcus digitalis*. Among patients with COVID-19, the gut microbiota exhibited an imbalance in the *Firmicutes/Bacteroidetes* ratio, while the proportion of *Proteobacteria* and *Actinomycetes* increased (Huang et al., 2023). Alterations in the gut microbiota can affect immune function, modulate gut-lung axis activity, and indirectly impair lung barrier function. Most current research primarily investigates gut microbiota alterations induced by lung diseases. However, a notable paucity exists regarding systematic quantitative data on the dynamic changes in the concentration, tissue distribution, and metabolic pathways of gut microbiota-derived metabolites, such as SCFAs, bile acids, and indoles, within the host. The underlying mechanisms and causal relationships necessitate extensive experimental validation to elucidate. Research investigating the impact of exercise on lung-related diseases via gut-lung axis modulation remains nascent, with a limited number of investigations. Evidence suggests that exercise can modulate the gut microbiota and its metabolites, thereby positively influencing the prevention, management, and treatment of lung diseases. Aerobic exercise can increase the abundance of SCFAs producing bacteria and modulate microbial structure, consequently elevating circulating SCFAs concentrations and exerting beneficial effects, including anti-inflammatory and immunomodulatory actions, to mitigate the risk of chronic obstructive pulmonary disease progression. Specifically, these benefits include improving depressive symptoms in patients with chronic obstructive pulmonary disease, enhancing respiratory function and exercise endurance, reducing the frequency of disease exacerbations, and balancing the body’s anti-inflammatory and pro-inflammatory responses (Kou et al., 2023). An 8-week experimental study involving aerobic exercise combined with a probiotic blend containing *Lactobacillus acidophilus*, *Lactobacillus bulgaricus*, *Bifidobacterium*, and *Streptococcus thermophilus* revealed that exercise can mitigate inflammatory responses, bolster resistance to upper respiratory tract infections, and enhance immune function (Saeedi et al., 2013). A systematic review expounded on how exercise can regulate the gut microbiota, inhibit inflammatory responses, maintain intestinal barrier homeostasis, and consequently modulate lung inflammation and homeostasis. Specifically, exercise can regulate oxidative stress, enhance the tight junction integrity between intestinal epithelial cells, modulate the composition and structure of the gut microbiota, and reduce oxidative stress markers. Furthermore, it improves intestinal barrier permeability, thereby preventing respiratory tract infections. Ultimately, it maintains gut microbial homeostasis and promotes healthy lung function (Yin et al., 2025). Collectively, these studies indicate that exercise can modulate respiratory system pathogenesis through gut microbiota regulation, suggesting that exercise can enhance gut microbial diversity, optimize its structure and function, inhibit inflammation, and maintain lung health, thereby ameliorating lung-related diseases. However, the specific impact of exercise types, intensity, duration, and frequency on the gut microbiota warrants further in-depth investigation (Table 3).

**Table 3:** Mechanistic pathways by which exercise modulates the gut microbiota in lung related diseases.

Disease type	Sports	Before intervention	After the intervention	Possible mechanisms of action
Asthma	Moderate intensity aerobic exercise	Disruption of the gut lung axis and gut microbiota dysbiosis may exacerbate airway inflammation.	Improved asthma control was observed, accompanied by a reduced frequency of exacerbations.	Aerobic exercise favorably modulates gut microbiota composition and increases the abundance of short chain fatty acid producing bacteria. Microbiota derived metabolites, including propionate and butyrate, enter the systemic circulation, inhibit T helper 2 immune responses, regulate pulmonary immune function, and thereby reduce airway inflammation (Andrade et al., 2014).
Chronic obstructive pulmonary	Aerobic exercise	Secondary gut microbiota dysbiosis	Increased muscle strength and mass were observed, accompanied by improved capacity for daily activities.	Aerobic training increases overall physical activity levels and promotes gut microbiota diversity. Microbiota derived may reduce systemic inflammation, thereby contributing to improved pulmonary function (Kou et al., 2023).

### 5.7 The association between gut microbiota and musculoskeletal diseases

The relationship between the gut microbiota and musculoskeletal diseases is reciprocal (Figure 10). On the one hand, ecological imbalance of the intestinal flora can lead to muscle atrophy and osteoporosis. On the other hand, musculoskeletal diseases may alter the host’s metabolic and immune status, thereby further influencing the composition and function of the intestinal microbiota as well as the production of intestinal metabolites (Hernandez, 2021). In 2012, the concept of the “gut–bone axis” was first proposed. Early studies primarily focused on alterations in the gut microbiota associated with musculoskeletal diseases, reporting a marked reduction in microbial diversity, an increased abundance of Bacteroidetes, and a decreased abundance of Firmicutes. Subsequent studies have emphasized the effects of gut microbiota-derived metabolites on skeletal muscle metabolism through inflammatory pathways, immune regulation, and neuroregulatory mechanisms. SCFAs, including acetic acid, propionic acid, and butyric acid, can stimulate the secretion of insulin-like growth factor-1, promote osteoblast activity, and inhibit osteoclast differentiation. Disruption of the intestinal barrier increases endotoxin levels and activates the TLR4–NF-κB signaling pathway, triggering chronic low-grade inflammation. This process accelerates bone resorption and muscle protein degradation, alters the Th17/Treg cell ratio, disrupts the RANKL/OPG balance, and ultimately leads to excessive osteoclast activation. Nevertheless, most existing studies have focused on populations in northern Europe and northern China, where pronounced regional and demographic differences make confounding variables difficult to control. In patients with osteoporosis, gut microbiota diversity is significantly reduced, accompanied by an imbalance between *Firmicutes* and *Bacteroides*. Reduced abundances of *Bacteroides*, *Arthrobacter*, *Megakrobacter*, and *anaerobic bacilli* impair short-chain fatty acid metabolism, thereby influencing host metabolic and immune processes as well as bone metabolism (Huang et al., 2022). Similarly, patients with muscle atrophy exhibit decreased intestinal microbial diversity, reduced abundance of *Clostridium*, and increased abundance of *Lactobacillus*, which may affect muscle function through alterations in protein metabolism, nutrient absorption, and inflammatory pathways (Kang et al., 2021). Collectively, these findings indicate the presence of a reciprocal axis between the gut microbiota and the musculoskeletal system. Interactions along the gut microbiota–muscle and gut microbiota–bone axes are associated with reduced microbial diversity, a lower proportion of beneficial bacteria, and an increased abundance of harmful bacteria, thereby elevating the risk of musculoskeletal diseases. From an exercise perspective, the association between the gut microbiota and the musculoskeletal system appears to occur relatively early. Both aerobic and resistance exercise can enhance microbial abundance and diversity and promote increases in bone mass. Long-term regular exercise lasting more than 6 weeks improves gut microbiota composition, whereas moderate- to low-intensity exercise is more favorable for the development of microbial diversity. In contrast, prolonged high-intensity exercise reduces the abundance of *Bifidobacterium*, which is detrimental to bone growth (Guo and Sun, 2023). After 14 weeks of moderate-intensity exercise, the Shannon index of the gut microbiota increases, body fat coefficients decrease, and the relative abundance of *Bifidobacterium*, *Faecalibacterium*, and *Phascolarctobacterium* increases, leading to improved exercise performance. These changes are associated with enhanced metabolism of glucose, flavonoids, arginine, and proline. Exercise-induced modulation of the gut microbiota is therefore thought to influence musculoskeletal diseases by altering core bacterial populations and their metabolic activity, thereby affecting skeletal muscle exercise capacity (Wang et al., 2021). In animal models, antibiotic-induced gut microbiota dysbiosis impairs skeletal muscle adaptability to exercise. In contrast, mice not exposed to antibiotics exhibit significant increases in muscle weight, fiber size, and fiber composition following exercise training. These findings demonstrate that gut microbiota imbalance weakens exercise-induced muscle hypertrophy and functional improvements, suggesting that a healthy gut microbiota composition is essential for maintaining skeletal muscle health and exercise adaptation (Tremblay et al., 2021). Overtraining and inappropriate dietary patterns can disrupt intestinal microbial balance, impair regulation of the gut–muscle axis, and exacerbate inflammation. Conversely, moderate exercise optimizes gut microbiota composition, modulates immune function to reduce oxidative stress, and promotes muscle protein synthesis, mitochondrial biogenesis, and glycogen storage. These benefits are observed not only in athletes but also in the general population engaged in physical training (Petriz et al., 2020). Overall, gut microbiota status influences athletic performance and post-exercise fatigue recovery, with underlying mechanisms related to enhanced antioxidant enzyme activity in physically active individuals (Hsu et al., 2015).

## 6 Possible molecular mechanisms by which exercise regulates the intestinal flora to mediate disease regulation

The potential molecular mechanisms through which exercise regulates the gut microbiota to influence disease processes primarily focus on those affecting pathological pathways. Currently, experimental studies investigating the effects of exercise duration and frequency on the gut microbiota during disease progression remain limited. Most studies are descriptive, examining potential correlations among exercise, the gut microbiota, and disease outcomes, while causal relationships are seldom investigated. This section examines the potential molecular mechanisms through which exercise regulates the gut microbiota to mediate disease outcomes, with a focus on different exercise types (Table 4). It integrates diseases not addressed in the previous section to explore how various exercise modalities influence the gut microbiota in diverse pathological conditions. In summary, the molecular mechanisms by which exercise regulates the gut microbiota primarily involve intestinal metabolites, immune regulation, inflammation modulation, and the gut–organ axis. These mechanisms contribute to maintaining homeostasis of the gut microbiota, preserving intestinal barrier integrity, enhancing intestinal epithelial immune cell function, reducing inflammatory marker expression, limiting interactions between pathogenic bacteria and host organs, and thereby aiding in disease prevention and treatment.

**Table 4:** Discussion on the mechanism of exercise regulating intestinal flora in diseases.

Disease type	Sports	Before intervention	After the intervention	Possible mechanisms of action
Inflammatory bowel disease, ibd	Moderate-intensity aerobic exercise	Decreased gut microbiota diversity, reduced abundance of short-chain fatty acid-producing bacteria, and impaired intestinal barrier integrity were observed.	Increased microbial diversity, elevated abundance of short-chain fatty acid-producing bacteria, improved intestinal barrier function, reduced stress levels, and alleviated intestinal symptoms were observed.	Aerobic exercise increases intestinal blood flow and peristalsis, thereby improving the intestinal microbial environment. These changes promote the proliferation of short chain fatty acid producing bacteria, such as *Actinobacillaceae*, leading to elevated butyrate concentrations and enhanced intestinal epithelial tight junction integrity. Consequently, activation of the NF-κB signaling pathway is inhibited, contributing to the attenuation of mucosal inflammation (Li et al., 2024).
	Yoga/Body–Mind Exercise			Exercise-induced cardiopulmonary responses modulate autonomic nervous system activity, thereby attenuating stress induced increases in intestinal permeability. These effects support the maintenance of microbial homeostasis and contribute to improvements in visceral hypersensitivity through interactions along the gut brain axis and the gut microbiota (Bower and Irwin, 2016).
	Resistance exercise			Resistance training increases skeletal muscle mass and improves systemic metabolic status, thereby indirectly reducing inflammatory stress within the intestinal environment. These adaptations create favorable conditions for recovery of the gut microbiota (Ordille and Phadtare, 2023).
	High-Intensity Interval Training			High-intensity interval training (HIIT) induces acute alterations in intestinal oxygenation and metabolic activity, which may rapidly modulate the metabolic product profile of the gut microbiota. These changes contribute to the development of systemic anti-inflammatory adaptations (Denou et al., 2016).
Urritable bowel syndrome	Regular aerobic exercise	Despite dysregulation of the gut–brain axis, gut microbiota imbalance, and reduced microbial diversity, the overall severity of symptoms was reduced, as evidenced by decreased abdominal pain and favorable changes in specific bacterial taxa.	The overall severity of symptoms was reduced, accompanied by decreased abdominal pain and favorable changes in specific bacterial taxa.	Regular aerobic exercise modulates autonomic nervous system balance, thereby improving intestinal motility and the gut internal environment. These changes promote the enrichment of beneficial bacterial taxa, while microbiota derived SCFAs and *γ* aminobutyric acid (GABA) contribute to the regulation of enteric neural activity and visceral sensation (Fani et al., 2019).
	Tai Ji			Taiji practice alleviates psychological stress, thereby disrupting the self reinforcing cycle between stress and gut microbiota dysbiosis. Through modulation of the gut brain axis, these effects contribute to restoration of microbiota balance and improvement of intestinal function (Hamasaki, 2017).
	Yoga			The integration of postural practices, breathing techniques, and mindfulness modulates hypothalamic–pituitary–adrenal (HPA) axis activity, thereby reducing the abundance of opportunistic pathogens such as *Klebsiella pneumoniae* in the gut and contributing to the restoration of microbial homeostasis (Chao et al., 2024).
Chronic renal failure	Aerobic exercise	Accumulation of uremic toxins is associated with severe gut microbiota dysbiosis.	The intervention alleviated symptoms of renal disease and reduced the accumulation of pathogenic bacteria.	Aerobic exercise improves cardiovascular function and systemic metabolic status, thereby partially alleviating intestinal damage induced by uremic toxins. These effects may reduce the production and accumulation of sulfated indole phenols, a class of gut microbiota–derived uremic toxins (Headley et al., 2020).
Stein-Leventhal syndrome	Resistance exercise	Gut microbiota dysbiosis, reduced skeletal muscle mass, and a low basal metabolic rate were observed.	Increased muscle strength and lean body mass, decreased body fat percentage and waist circumference, and improved insulin sensitivity	Resistance training increases skeletal muscle mass and basal metabolic rate, thereby enhancing systemic glucose and lipid metabolic capacity. These metabolic improvements promote favorable alterations in gut microbial community structure, while microbiota derived metabolites are involved in the regulation of sex hormone homeostasis (Nasiri et al., 2025).
	Aerobic exercise			Aerobic exercise increases glucose utilization, thereby contributing to stabilization of the gut microbiota and maintenance of microbial homeostasis. These changes support regulation of intestinal immune function and beneficial modulation of autoimmune responses (Leng et al., 2023).
	Aerobic combined with resistance training			Joint training exerts synergistic effects on metabolic regulation and sex hormone homeostasis through microbiota derived metabolites, including and bile acids (Nasiri et al., 2025).
I type 2 diabetes mellitus	Resistance exercise	The condition was characterized by gut microbiota dysbiosis.	A reduction in daily insulin dose was observed.	Resistance training increases skeletal muscle glycogen storage, thereby attenuating stress induced damage to the intestinal mucosa and gut microbiota associated with fluctuations in blood glucose levels (de Abreu Lima et al., 2022).
II type 2 diabetes mellitus	Aerobic combined with resistance exercise	Lipid metabolic dysfunction, gut microbiota dysbiosis, and elevated blood glucose levels were observed.	Hypoglycemia	Joint training improves systemic glucose and lipid metabolism, thereby contributing to restoration of gut microbiota homeostasis. The restored microbiota, through the production of SCFAs, supports regulation of blood glucose levels (Torquati et al., 2023).
Alzheimer’s disease	Aerobic exercise	High levels of psychological stress and a high prevalence of anxiety and depression were observed.	Improved sleep quality and reduced disease activity were observed.	Aerobic exercise increases cerebral blood flow and brain derived neurotrophic factor (BDNF) levels, thereby improving brain metabolic function. These central adaptations are accompanied by modulation of gut microbiota composition. Microbiota derived SCFAs can access the central nervous system via the blood brain barrier, where they contribute to inhibition of neuroinflammation and promotion of synaptic plasticity (Wei et al., 2025).
	Resistance exercise	Intracerebral β-amyloid deposition, pronounced neuroinflammation, and gut microbiota dysbiosis were observed.	The rate of cognitive function decline was attenuated, accompanied by improvements in memory and executive function.	Resistance training maintains skeletal muscle mass and basal metabolic rate, thereby promoting intestinal blood circulation and mucosal health. These adaptations contribute to enhanced blood–brain barrier integrity and improved neuroimmune function (Qaisar et al., 2024).
Parkinson’s disease	Taiji	Marked gait instability and balance impairment were observed, accompanied by an increased risk of falls.	Improved gait stability, enhanced balance performance, and a reduced incidence of falls were observed.	Tai ji practice activates cerebellar–basal ganglia pathways, leading to modulation of autonomic nervous system function and optimization of intestinal motility. These effects promote gut microbial balance and are associated with modulation of dopamine neurotransmitter metabolism (Li et al., 2025).
Autism Spectrum Disorder	Structured aerobic exercise	Social communication disorders, pronounced stereotyped behaviors, and gut microbiota dysbiosis were observed.	Reduced stereotyped behaviors, prolonged attention span, and improvements in emotional and behavioral symptoms were observed.	Regular aerobic exercise modulates autonomic nervous system balance, thereby improving the intestinal environment and gut microbiota composition. Microbiota-derived metabolites, including SCFAs and γ-aminobutyric acid (GABA), may influence central nervous system excitability and brain regions associated with social behavior via vagal signaling or systemic circulation (Ma, 2021).

### 6.1 The role of metabolites

#### 6.1.1 Short-chain fatty acids (SCFAs)

SCFAs play a crucial role in regulating host metabolism, maintaining intestinal barrier function, inhibiting inflammatory responses, and improving insulin sensitivity. The metabolites of the gut microbiota SCFAs mainly exert their metabolic regulatory effects through two primary mechanisms: inhibition of histone deacetylases to regulate gene expression andsignaling through G protein-coupled receptors GPR41 and GPR43. SCFAs also maintain intestinal barrier function and reduce inflammatory responses (Kang et al., 2021). SCFAs activate the AMPK pathway, thereby promoting mitochondrial biogenesis and enhancing the efficiency of fatty acid oxidation and energy metabolism. Exercise not only optimizes the composition of the intestinal microbiota by increasing the abundance of SCFA-producing bacteria, but also promotes the production and release of SCFAs. Aerobic exercise, resistance training, and high-intensity interval training can significantly increase intestinal SCFA levels, thereby improving the metabolic status of patients with type 2 diabetes (Liu and Qiu, 2023b). Furthermore, exercise promotes fatty acid oxidation and mitochondrial biogenesis by regulating the SCFA-mediated AMPK pathway, thus further improving insulin sensitivity (Liu and Qiu, 2023a). Additionally, exercise can alter the intestinal environment by increasing oxygenation and lowering the pH value, thereby promoting the colonization of beneficial bacteria and the production of SCFAs (Liu and Peng, 2025). Much of the current evidence for the influence of SCFAs on histone deacetylases and GPR41/43 is derived mainly from *in vitro* and animal experiments. The causal relationships underlying the metabolic pathways of SCFAs in the human gut microbiota remain largely inferential. The activation and metabolic improvement of the AMPK pathway induced by SCFAs have been validated across multiple animal and human exercise interventions. The causal inference for this pathway is stronger than that for the other AMPK-related mechanisms. In conclusion, the overall causal chain linking exercise, SCFAs, and metabolism is relatively well-established in animal models; however, direct evidence from long-term human studies remains limited, and larger-scale randomized controlled trials are needed to confirm these findings.

#### 6.1.2 Tryptophan metabolites

Exercise can significantly alter the composition of the gut microbiota and affect the production of tryptophan metabolites. Dysbiosis of the gut microbiota in high-fat diet-induced animal models disrupts the tryptophan metabolic pathway and significantly reduces the production of 5-hydroxyindole-3-acetic acid (5-HIAA) (Du et al., 2024). Exercise enhances the capacity of the gut microbiota to metabolize tryptophan and promotes the production of indole-3-propionic acid and 5-HIAA, thereby improving metabolic health (Scheiman et al., 2019). Indole-3-propionic acid activates aryl hydrocarbon receptors (AhR) to enhance intestinal barrier function and reduce intestinal permeability. 5-HIAA improves insulin signaling in the liver through the AhR/TSC2/mTORC1 axis, thereby reducing the risk of type 2 diabetes and alleviating its progression. Exercise regulates the gut microbiota, enhances intestinal barrier function, and reduces inflammatory responses (Du et al., 2024). A cross-sectional study in patients with type 2 diabetes found that their 5-HIAA levels were significantly lower than those in healthy individuals, suggesting that 5-HIAA may serve as a potential therapeutic target and early diagnostic biomarker for such patients with type 2 diabetes. This discovery provides a new direction for the development of diabetes intervention measures based on gut microbiota metabolites. The current research on the mechanism by which the gut microbiota influences host physiology through tryptophan metabolites has been supported in large-scale animal experiments and small-sample clinical interventions. In animal experiments, the use of aseptic environments or pharmacological blockade of irrelevant variables can effectively control confounding factors. Therefore, animal studies are more convincing in terms of mechanism clarification and causal verification. Human data, however, are essential for large-scale cohort studies and clinical trials to validate associations and have greater relevance to the serum expression of 5-HIAA. Moreover, recent Mendelian randomization studies and multi-omics analyses have further supported the causal association between 5-HIAA levels and type 2 diabetes. However, there remains a lack of large-scale human randomized controlled trials and longitudinal studies to elucidate this mechanistic pathway, particularly the acute and long-term effects of exercise on 5-HIAA levels, as well as potential interactions between SCFAs and tryptophan metabolites influenced by the gut microbiota. Currently, no studies have demonstrated such interactions between these two pathways.

#### 6.1.3 Bile acid metabolism

Bile acids play a crucial role in exercise-regulated gut microbiota metabolism, lipid metabolism, and cardiovascular health (Table 2). Exercise modulates the gut microbiota, thereby promoting the production of secondary bile acids, activating FXR (Wang et al., 2020), enhancing GLP-1 secretion, and exerting protective effects on articular cartilage (Jin and Su, 2023). Exercise alters gut microbiota composition, increasing the relative abundance of *Firmicutes* and thereby enhancing bile acid metabolism. Furthermore, the gut microbiota converts primary bile acids into secondary bile acids via specific metabolic pathways, thereby influencing host energy metabolism (Tang et al., 2025). Bile acids regulate lipid metabolism primarily through the FXR-FGF15/19 signaling pathway, thereby reducing the risk of cardiovascular diseases. These combined mechanisms underscore the pivotal role of bile acids in exercise-mediated regulation of metabolic health (Xia et al., 2024). Prolonged exercise has been shown to decrease bile acid levels in both serum and feces, suggesting that exercise may directly enhance bile acid excretion, thereby modulating lipid metabolism and ameliorating obesity. This pattern of change has also been observed in animal models even when body weight changes were not significant (Farahnak et al., 2017). Therefore, the present review proposes that exercise exerts a direct beneficial effect on bile acid metabolism. Subsequently, by modulating other metabolic pathways, it attenuates the progression of various human diseases. In conclusion, research on the regulation of bile acid metabolic pathways has progressed from molecular-level investigations to systemic mechanistic studies, with consistent findings validated in both animal models and human pharmacological studies. However, the potential influence of confounding factors on these benefits has not yet been fully excluded, and the precise pathway linking exercise, bile acids, and metabolic improvement remains to be fully elucidated (see Table 5).

**Table 5:** Bile acid metabolic pathways modulated by the gut microbiota.

Incident	Summary
The gut microbiota converts primary bile acids into secondary bile acids (Ty et al., 2022).	Scientific studies have shown that intestinal flora (such as *Bacteroides* and *Clostridium*) convert primary bile acids synthesized by the liver into secondary bile acids by secreting enzymes such as bile salt hydrolase.
Bile acids regulate lipid metabolism via the FXR-FGF 15 pathway (Wu et al., 2021).	Bile acids, particularly secondary bile acids, regulate lipid metabolism by activating FXR, which in turn induces FGF-15 expression. This subsequently inhibits the expression of cholesterol 7α-hydroxylase, thereby reducing bile acid synthesis.
Bile acids improve metabolic health by activating TGR5 receptors (Wahlström et al., 2016).	Subordinate bile acids primarily exert beneficial effects on host metabolic health by activating the G protein-coupled receptor TGR5, thereby enhancing energy expenditure, promoting fatty acid oxidation, and improving insulin sensitivity and glucose homeostasis.
There is a bidirectional regulatory relationship between intestinal flora and bile acids (Zeng et al., 2021).	Research shows that gut microbiota not only alters the composition and levels of bile acids through metabolic processes, but bile acids can also regulate their synthesis and metabolism, as well as the structure and function of gut microbiota, by activating the FXR receptor.
The imbalance of bile acid metabolism is related to various metabolic disorders (He et al., 2023).	The study found that the ecological imbalance of intestinal flora can disrupt the metabolism of bile acids, leading to abnormal composition of bile acids. This is closely related to the occurrence and progression of various metabolic disorders, including obesity, diabetes and nonalcoholic fatty liver.

### 6.2 Immune regulation and inflammatory regulation

Exercise prevents and ameliorates various obesity-related chronic inflammatory diseases, including those associated with diabetes, cardiovascular disease, and inflammatory bowel disease, through multiple mechanisms: optimization of intestinal microbiota composition, enhanced production of SCFAs, suppression of NF-κB signaling, modulation of immune cell function, and regulation of the gut-brain axis that influences immune regulation. Exercise modulates intestinal microbiota composition by decreasing pro-inflammatory bacteria and increasing certain anti-inflammatory taxa, thereby attenuating systemic inflammation. It also promotes SCFAs production by the intestinal microbiota, elevating the concentrations of butyrate, propionate, and acetate. These SCFAs inhibit histone deacetylases, modulate immune cell function, suppress the expression of pro-inflammatory cytokines, and enhance the secretion of anti-inflammatory factors. High-intensity interval training has been shown to decrease serum levels of the pro-inflammatory cytokines IL-6 and TNF-*α* by approximately 30%, while simultaneously elevating the anti-inflammatory cytokine IL-10. Moreover, SCFAs mitigate inflammatory diseases, such as inflammatory bowel disease, by inhibiting the NF-κB signaling pathway and reducing the release of inflammatory mediators. Randomized experiments comparing exercise and non-exercise groups have demonstrated significantly elevated butyrate levels and markedly reduced pro-inflammatory cytokines TNF-α and IL-6 in the exercise group (Huang et al., 2013). Exercise further attenuates inflammation by altering gut microbiota composition—increasing beneficial taxa while decreasing harmful ones and indirectly by regulating neural function via the gut-brain axis, thereby influencing emotional and cognitive states. Additionally, exercise-induced endogenous cannabinoids exert anti-inflammatory effects and reduce the risk of inflammatory diseases by modulating the immune system and metabolic pathways. In conclusion, both human pathological evidence and animal studies have established causal relationships between exercise-regulated gut microbiota alterations and modulation of immune and inflammatory pathways. Future research, particularly large-scale randomized controlled trials, is warranted to elucidate the overlapping mechanisms of immune regulation and inflammatory modulation, thereby advancing our understanding of disease pathogenesis and facilitating improved therapeutic strategies.

### 6.3 Gut–organ Axis

#### 6.3.1 Gut–muscle Axis

**Figure 11:**
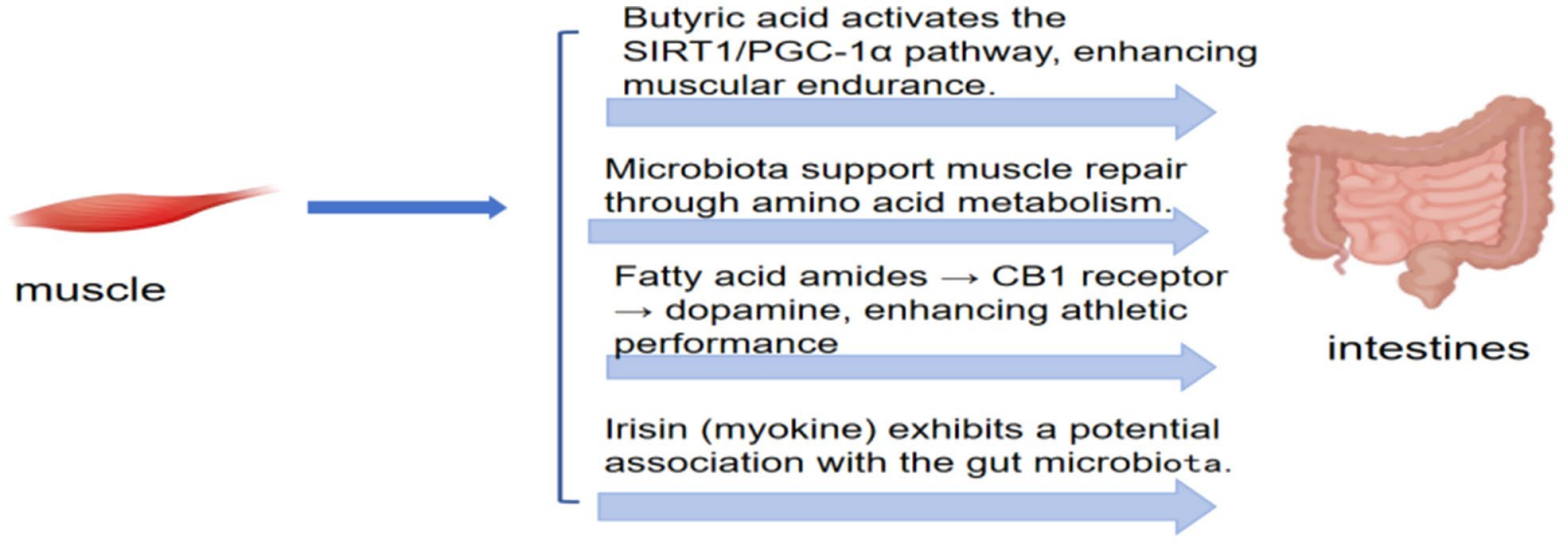
Mechanistic interactions between the gut–muscle axis and the gut microbiota.

The gut muscle axis describes bidirectional interactions between the gut microbiota and skeletal muscle tissue (Figure 11). Exercise modulates muscle function and metabolism partly by reshaping the gut microbiota. Exercise-enriched taxa, including *Lactobacillus* and *Eisenbergiella*, are positively associated with muscle enzyme activity (alanine aminopeptidase) and may support muscle repair by promoting amino acid metabolism. The gut microbiota may promote postexercise recovery and muscle health by modulating metabolic functions in muscle tissue (Gao and Zhang, 2024). Butyrate, a metabolite derived from the gut microbiota, activates the SIRT1–PGC-1α pathway, promotes oxidative metabolism in slow twitch muscle fibers, and enhances muscle endurance. The SIRT1–PGC-1α pathway plays a key role in energy metabolism; its activation enhances mitochondrial function and improves muscle endurance and recovery capacity (Zhang, 2014). Exercise reshapes the gut microbiota and enhances the production of fatty acid amides by bacteria such as *Eubacterium rectale* and *Coprococcus eutactus*. These metabolites activate intestinal cannabinoid 1 receptors, increase dopamine levels in the ventral striatum, and improve exercise performance (Dohnalová et al., 2022). Irisin, an important myokine, improves lipid metabolism (Aladag et al., 2023); however, its interactions with the gut microbiota remain unclear. Nevertheless, a close connection cannot be ruled out. In summary, the gut muscle axis has evolved from a conceptual stage to a more integrated network encompassing multiple mediators, including SCFAs, AMPK signaling, the SIRT1–PGC-1α pathway, endocannabinoids, dopamine, and irisin. Among these, signaling involving SCFAs, SIRT1–PGC-1α, and AMPK has the most extensive support in animal studies; several human metabolic studies also suggest beneficial effects on insulin sensitivity. Compared with nonmodifiable factors such as age and hormonal status, the gut muscle axis is modifiable, providing unique value for intervention. Combined strategies integrating exercise, diet, and probiotics may achieve synergistic effects.

#### 6.3.2 Gut–brain Axis

**Figure 12:**
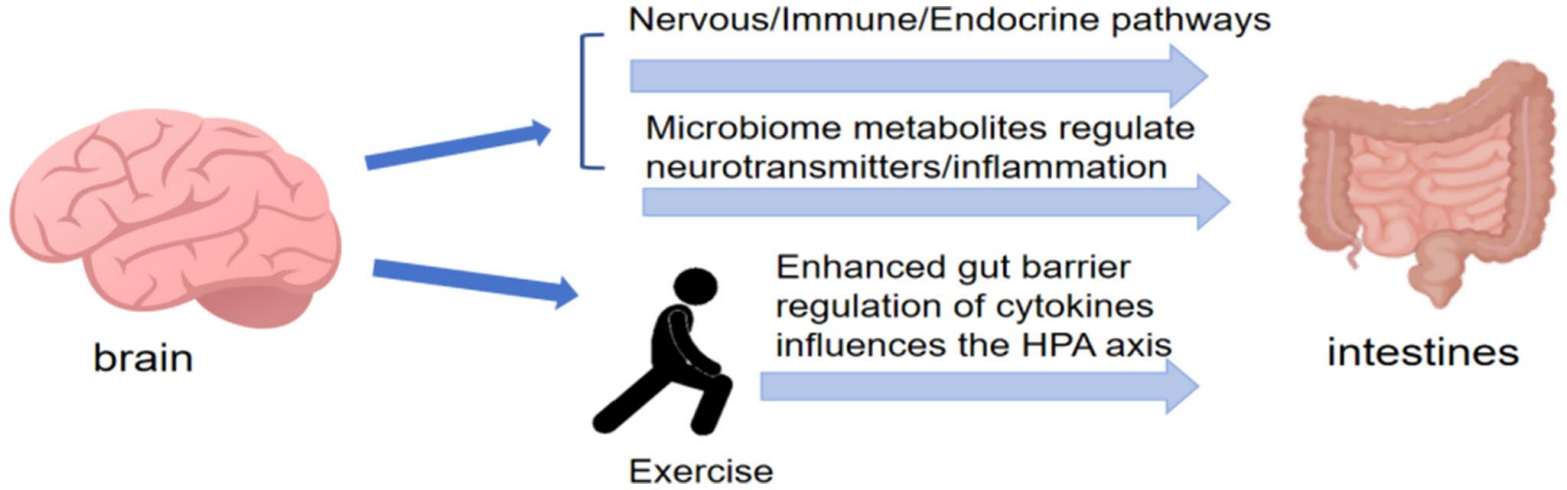
Mechanistic interactions between the gut–brain axis and the gut microbiota.

The gut brain axis refers to a bidirectional communication network between the intestine and the brain, mediated through neural, immune, and endocrine pathways. Gut microbiota derived metabolites, including SCFAs and tryptophan, affect brain function through the vagus nerve and systemic circulation. Butyrate attenuates microglial activation and inhibits neuroinflammation. In addition, 5-HIAA improves depressive symptoms by modulating serotonin synthesis (Bravo et al., 2011). Communication within the gut brain axis involves various metabolites crossing the blood brain barrier, beyond direct neurotransmitter action, to regulate neurotransmitter synthesis and release, thereby influencing mood, cognition, and behavior (Figure 12). SCFAs mediate gut brain axis communication by activating G protein coupled receptors, thereby stimulating intestinal endocrine cells to secrete peptide YY, glucagon like peptide 1, and serotonin (Wang et al., 2025). Bidirectional regulation of the gut brain axis plays a crucial role in various neurological disorders, including depression, anxiety disorders, and autism spectrum disorders. Dysbiosis of the intestinal microbiota leads to neurotransmitter imbalance, which in turn contributes to the development of these disorders. Exercise enhances intestinal barrier function, reduces the release of inflammatory mediators, and attenuates neuroinflammation. It inhibits the expression of pro-inflammatory cytokines (TNF-*α* and IL-17) and increases the secretion of anti-inflammatory cytokines (IL-10), thereby improving the neuroinflammatory state. Exercise regulates the hypothalamic pituitary adrenal axis, triggering the release of adrenaline, norepinephrine, and glucocorticoids. These hormonal changes affect gastric acid secretion and intestinal mucus composition, thereby influencing the intestinal microbiota (Wang et al., 2025). In conclusion, exercise mediated regulation of the gut brain axis induces alterations in neural pathways and ameliorates disease related pathophysiological processes in neurological disorders. However, although 5-HIAA holds a central position in traditional depression models, its direct causal relationship with the gut brain axis remains unclear and has not yet been confirmed in large scale human studies.

#### 6.3.3 Gut–liver axis

**Figure 13:**
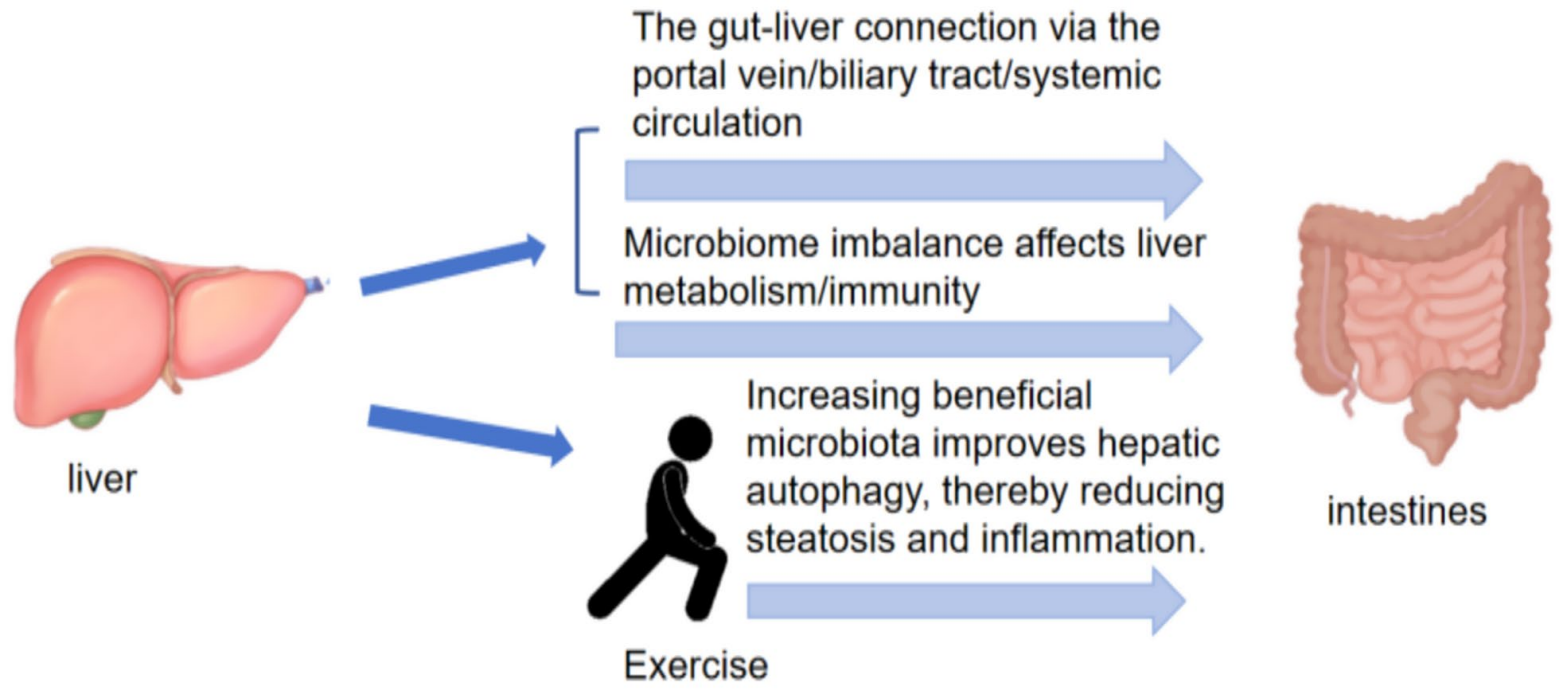
Mechanistic interactions between the gut–liver axis and the gut microbiota.

The gut liver axis refers to the close and dynamic interaction between the intestine and the liver. Exercise affects liver metabolism and immune function by regulating the intestinal microbiota and improving intestinal barrier function (Figure 13). Exercise also plays a significant role in the management of liver diseases, including nonalcoholic fatty liver disease and metabolism related fatty liver disease. In experimental models, exercise increased the diversity and abundance of beneficial microbiota in mice with nonalcoholic fatty liver disease, enhanced hepatic autophagy, and reduced liver steatosis and inflammation (Yu et al., 2024). Exercise regulates the intestinal microbiota and reduces levels of pro-inflammatory mediators, thereby protecting the liver from injury (Tamura et al., 2023). The intestine and liver are interconnected through the portal vein, biliary tract, and systemic circulation, forming a complex dynamic system. The intestine serves as the primary site for nutrient absorption, whereas the liver performs key functions, including metabolism, detoxification, and bile secretion. As an important component of the gut microbiome, microbial dysbiosis directly affects liver health (Liu and Qiu, 2023b). Exercise affects the gut microbiota through multiple mechanisms, including the gut brain axis, gut muscle axis, and metabolic by products, thereby regulating overall health. This process involves multiple pathways, including the intestinal environment, intestinal barrier function, intestinal microbiota composition, and oxidative stress. In conclusion, exercise mediated regulation of the intestinal microbiota for disease prevention and management is achieved through the gut liver axis. Both animal experiments and human cross sectional studies have demonstrated that exercise can restore intestinal microbiota diversity. In addition, exercise can repair the intestinal barrier and reduce endotoxin translocation into the systemic circulation. However, the causal roles of SCFAs, inflammation immune regulation, and portal vein mediated metabolic pathways remain to be experimentally validated. Although metabolomic analyses have revealed associations between metabolites such as choline and nonalcoholic fatty liver disease, whether these metabolites act as direct pathogenic factors has not been confirmed by gene editing or pharmacological antagonism studies. Moreover, potential interactions with the gut brain axis or gut muscle axis have not yet been conclusively demonstrated.

## 7 Summary and outlook

Current research systematically reveals the intricate interactions among the gut microbiota, exercise, and disease. As the “second genome” of humans, the gut microbiota profoundly influences disease onset and progression through metabolic, immune, and neuroendocrine pathways. Ecological dysbiosis reduces beneficial metabolites while increasing harmful components and is closely associated with metabolic, immune, and neurological disorders, as well as other conditions, including traumatic injury, dermatological diseases, ocular diseases, liver diseases, cardiovascular disorders, pulmonary diseases, and musculoskeletal disorders.

Exercise and the gut microbiota exhibit bidirectional regulatory interactions. The gut microbiota influences muscle metabolism, exercise capacity, and physical adaptation through pathways such as the gut muscle axis. Conversely, exercise reshapes the gut microbiota by altering its composition, regulating microbial function, and improving the intestinal environment. The effects of exercise vary according to its type, intensity, and duration. Moderate intensity aerobic exercise is more conducive to microbial homeostasis, whereas excessive exercise, in which physiological adaptation exceeds recovery capacity, may exert adverse effects. Further evidence indicates that exercise modulates disease processes through metabolites such as, tryptophan metabolites, and bile acids, as well as through immune regulation and multiple cross organ axes, including the gut muscle, gut brain, and gut liver axes. These findings provide novel mechanistic insights for disease prevention and management.

A major research gap remains in that the causal relationships between the gut microbiota and disease related physiological mechanisms have not been fully established. In human studies, pathological investigations of the gut microbiota are challenged by confounding variables related to microbial composition and other target organs, making it difficult to determine whether microbial alterations are causal or consequential. Therefore, future research should focus on molecular mechanisms to clarify the pathways linking exercise, gut microbiota metabolism, and disease, thereby facilitating the development of precisely targeted therapeutic strategies. In addition, studies examining the type and duration of exercise interventions remain limited, with most evidence derived from animal models. Future research should include large scale, in depth investigations of longitudinal changes in the human gut microbiota. Moreover, individual variability in response to exercise interventions should be carefully considered to determine appropriate exercise type, intensity, and duration for different populations, thereby optimizing intervention strategies. At the same time, clinical translational research should be strengthened to promote integrated therapeutic approaches combining exercise mediated modulation of the gut microbiota with probiotic and dietary interventions, thereby providing more precise and effective strategies for disease prevention and management.
